# Development of a Digital Tool for People With a Long-Term Condition Using Stroke as a Case Example: Participatory Design Approach

**DOI:** 10.2196/35478

**Published:** 2022-06-03

**Authors:** Emma K Kjörk, Katharina S Sunnerhagen, Åsa Lundgren-Nilsson, Anders K Andersson, Gunnel Carlsson

**Affiliations:** 1 Department of Clinical Neuroscience, Institute of Neuroscience and Physiology, Sahlgrenska Academy University of Gothenburg Gothenburg Sweden; 2 Sahlgrenska University Hospital Gothenburg Sweden; 3 Stroke Association Gothenburg Sweden

**Keywords:** eHealth, digital tool, Strokehälsa, follow-up, chronic care, stroke, Post-Stroke Checklist, health literacy, co-design, shared decision making

## Abstract

**Background:**

In patient care, demand is growing for digital health tools to enable remote services and enhance patient involvement. People with chronic conditions often have multiple health problems, and long-term follow-up is recommended to meet their needs and enable access to appropriate support. A digital tool for previsit preparation could enhance time efficiency and guide the conversation during the visit toward the patient’s priorities.

**Objective:**

This study aims to develop a digital previsit tool and explore potential end user’s perceptions, using a participatory approach with stroke as a case example.

**Methods:**

The digital tool was developed and prototyped according to service design principles, informed by qualitative participant data and feedback from an expert panel. All features were processed in workshops with a team that included a patient partner. The resulting tool presented questions about health problems and health information. Study participants were people with stroke recruited from an outpatient clinic and patient organizations in Sweden. Development and data collection were conducted in parallel. For conceptualization, the initial prototype was based on the Post-Stroke Checklist and research. Needs and relevance were explored in focus groups, and we used a web survey and individual interviews to explore perceived utility, ease of use, and acceptance. Data were thematically analyzed following the Framework Method.

**Results:**

The development process included 22 participants (9 women) with a median age of 59 (range 42-83) years and a median of 51 (range 4-228) months since stroke. Participants were satisfied or very satisfied with using the tool and recommended its use in clinical practice. Three main themes were constructed based on focus group data (n=12) and interviews (n=10). First, valuable accessible information illuminated the need for information to confirm experiences, facilitate responses, and invite engagement in their care. Amendments to the information in turn reconfigured their expectations. Second, utility and complexity in answering confirmed that the questions were relevant and comprehensible. Some participants perceived the answer options as limiting and suggested additional space for free text. Third, capturing needs and value of the tool highlighted the tool’s potential to identify health problems and the importance of encouraging further dialog. The resulting digital tool, Strokehälsa [Strokehealth] version 1.0, is now incorporated into a national health platform.

**Conclusions:**

The participatory approach to tool development yielded a previsit digital tool that the study group perceived as useful. The holistic development process used here, which integrated health information, validated questions, and digital functionality, offers an example that could be applicable in the context of other long-term conditions. Beyond its potential to identify care needs, the tool offers information that confirms experiences and supports answering the questions in the tool. The tool is freely shared for adaptation in different contexts.

**Trial Registration:**

researchweb 236341; https://www.researchweb.org/is/vgr/project/236341

## Introduction

The desire to encourage patient involvement [1] and the growing acceptance of digitized health care have contributed to a rising interest in digital health tools [2,3]. For people living with long-term conditions [4] and chronic disability [5], recurrent interventions and health care support are crucial. Stroke, which serves as the context of this study, is a common cause of disability, with more than 101 million cases worldwide in 2019 [6], and is associated with motor impairments and cognitive, emotional, and communication difficulties [5]. Organized systems of care, including follow-up and self-management, are beneficial for people with long-term conditions [4,7], particularly because they can have more difficulty in actively engaging with health services [8-10]. A redesign of health care services to ensure a prepared patient and a proactive health care team is crucial [4]. Digital health solutions may offer tools that can facilitate improved follow-up.

Digitizing has already been shown to speed up the redesign of health care [3] and potentially foster access to health care services [7,11]. In Sweden, the digital platform *Healthcare Guide 1177* [12], accessible to registered individuals, is widely used. The platform includes medical information and health tools such as previsit forms. However, regardless of the mode of service delivery, patients need person-centered support [1] and accessible health information [10]. Thus, digital tool design should take into consideration patient need for information [13] and the best ways to promote active engagement of patients in long-term care with their health professionals [2].

The use of previsit tools can make people feel more knowledgeable, better informed, and clearer about their values [14]. Moreover, digital health tools can enhance dialog with health professionals [15] and empower patients to become active partners [2]. A recent randomized clinical trial showed that a previsit digital tool for collecting contextual data from patients had a positive impact on patient-provider communication [16]. However, previsit digital tools often focus on collecting data but do not include health information for the patient [12,16,17], even though information is key to eHealth literacy [10], comprising a patient’s ability to understand, access, and use eHealth technologies. Patients need to be involved in self-management and interactions with health professionals [9,18], and digital solutions must be designed to promote eHealth literacy [10], counteract inequalities [18], and enhance shared decision making [19]. A thorough design process is indispensable to achieving this aim.

Service design is a human-centered approach that focuses on understanding the patient experiences to achieve a holistic view of solutions to complex problems [19,20]. Different methods can be applied, such as a “persona” that represents a member of a future user group, or a set of prototypes that offer alternative solutions for a digital tool [20,21]. Qualitative research often can be used to explore the needs of patients and health care providers before pilot versions of such tools are tested [20] or incorporated into secure health platforms for use. A combination of service design and co-design approaches is beneficial for understanding users’ needs in terms of technologies or processes [22]. In participatory co-design approaches, stakeholders—such as researchers, patients, and health care staff—work together throughout the design process [23]. The Technological Acceptance Model (TAM) [24] illustrates factors influencing adoption of technology and how perceived usefulness and ease of use affect acceptability.

People with long-term conditions need digital tools designed to cover a range of health problems and related information. Although previsit digital tools have been designed for people with various conditions [2,16,17,25], tools related to organized follow-up after stroke are scarce [11]. Furthermore, when digital elements are used, they are commonly part of a comprehensive and complex approach to poststroke follow-up [26,27] and lack a thorough description of the development process including user experiences [26]. Additionally, tools commonly request patient-reported data [16,17] without a combined solution in which patients in turn receive tailored health information. To our knowledge, no user-friendly previsit digital tool is yet available that includes well-validated self-report and health information to prepare people with stroke for a follow-up visit. Our aim was to develop a digital previsit tool and explore potential end user’s perceptions prior to testing it in a clinical setting, using a participatory approach with stroke as a case example.

## Methods

### Overview

A participatory [23] and pragmatic approach including mixed methods [28] was used to design a digital tool that meets users’ needs. In a participatory co-design approach, end users are viewed as experts on their experiences, and they can be engaged at different levels, with some becoming partners in the research team [23,29]. To ensure patient involvement, a patient with stroke who was engaged in a support association and had a background in information technology projects became a patient partner and coauthor. Initially, this patient partner (AKA) provided valuable advice regarding recruitment, participant involvement, and how to introduce the prototype. He reviewed the tool content and was involved in workshops with members of the research team (EKK, GC, and KSS). He also reviewed the summaries of the preliminary themes.

### Participants and Recruitment

Participants were included in 2 phases between December 2017 and October 2020. Staff identified eligible individuals in 3 settings: an outpatient unit, Stroke Forum (a center for support and advice after initial care), and a support association. Potential participants were briefly informed about the study, and those who agreed to participate were contacted by a researcher (EKK) via phone to provide detailed information. After purposive sampling with an attempt to achieve heterogeneity in terms of age, sex, communication, and mobility, participants were scheduled for an interview. They also were sent study information by email, including web links to a pilot version of the tool and to a web survey for the amendment phase. The inclusion criterion was having had a stroke. The exclusion criterion was severe communication or cognitive difficulties that made participation impossible, even in a small group discussion or together with next of kin. The sample size was guided by the concept of information power to enhance the richness of data according to the aim [30]. All participants gave their informed consent before data collection.

Members of the expert panel were contacted by the first author throughout the process during 2017-2020. Potential members were purposively recruited to represent different services, including members of the stroke association and health care professionals. Expert panel members received the link to the second pilot version and a separate MS Word document with the text included in the tool. Written feedback on text revisions was collected via email, unless verbal input was preferred. Members of relevant professions then were approached for specific feedback when appropriate. The expert panel (n=11, 3 males) had a median age of 55 years (range 42-70 years) and represented the following competencies: nurse (n=1), occupational therapist (n=3), physician (n=1), physiotherapist (n=1), neuropsychologist (n=1), speech therapist (n=2), service designer (n=1), and patient partner (n=1). Professionals had a median of 20 years (range 10-40 years) of stroke experience with the following education levels: doctoral degree (PhD; n=2), PhD student (n=3), and master’s degree (n=4).

### Development and Data Collection

**Figure 1:**
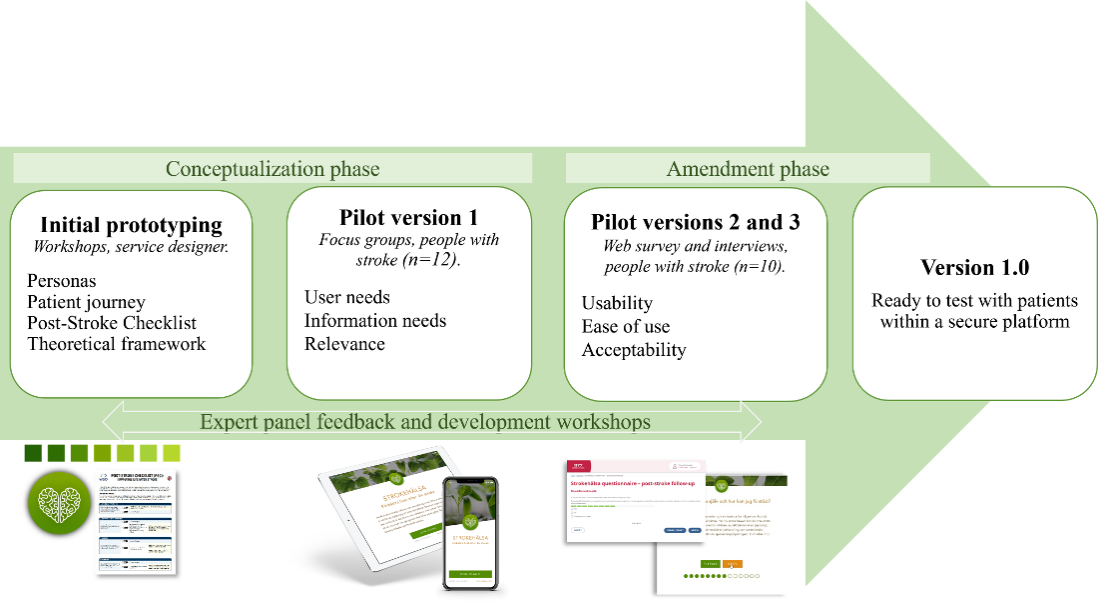
The different sources for input and visualization of prototypes used in the conceptual and amendment phases of the design process.

The co-design process integrated the development of the tool and the data collection, including user experiences. The process was performed in 2 phases: the conceptualization phase and the amendment phase, including a variety of methods involving different stakeholders (Figure 1).

### Development

The starting point for the conceptual phase comprised previous research regarding follow-up and suggestions for a patient version of the Post-Stroke Checklist (PSC) [31]. It was specified in advance that a digital previsit tool should be developed based on the PSC. Existing research related to stroke and person-centered care informed this development to ensure an evidence-based tool. The initial prototyping workshops were conducted by a service designer and the first author (EKK). The PSC [31,32], which constituted the basis for mapping the “patient journey” [20], is an easy tool for identifying common health problems and facilitating further actions, such as referrals to health services or patient organizations. It comprises 11 questions within the following areas: secondary prevention, activities of daily living, mobility, spasticity, pain, incontinence, communication, mood, cognition, life after stroke, and relationship with family. One example of a question is, “Since your stroke or last assessment, are you finding it more difficult to communicate with others?” The PSC is available from the World Stroke Organization and free for anyone to use. To foster an understanding of the potential user group, “personas” [20] were developed to represent users of different sexes, ages, personalities, life situations, values, and interests.

The content of and amendments to the tool were discussed during formal workshops and in dialogs with the research team, including the patient partner (EKK, GC, KSS, and AKA). These workshops guided the design of new pilot versions, and decisions were taken in consensus. Decisions were based on the data collection, expert panel feedback, and relevant evidence, and addressed, for example, clarification of the language by the addition or removal of text and answer options. All relevant data were combined into a single document before being systematically discussed in the workshops.

### Data Collection: User Needs and Experiences

During the conceptualization phase from December 2017 to March 2019, focus group discussions [33] were conducted within each location to explore user needs and the perceived relevance of an early prototype, known as pilot 1. Participants tested the pilot in their home environment before the focus group took place. In all groups, an interview guide [33] with open-ended questions was used (Multimedia Appendix 1), and the moderator gave a summary at the end, leaving open the possibility of correcting potential misunderstandings. Each focus group was conducted face-to-face, lasted approximately 60-90 minutes, and was audio recorded and transcribed verbatim. The focus group data and field notes informed further amendments and prototyping.

In the amendment phase from September to October 2020, pilot versions 2 and 3 were tested before data collection. Participants completed a web survey, followed by an individual interview, to explore perceived usefulness, ease of use, and acceptability, as inspired by the TAM [24]. The survey included previous web habits, demographic data, and satisfaction ratings, as follows: very satisfied, satisfied, dissatisfied, very dissatisfied, and don’t know. Along with the interview, self-reported characteristics were collected, including stroke type, time since stroke, and level of dependence in activities of daily living. The interviews were conducted by the first author (EKK) via phone, who followed an interview guide (Multimedia Appendix 1). During the interview, participants did “think aloud” [34] as they were using the tool. Support from next of kin was allowed during data collection.

### Analysis

Substantive cross-sectional data analysis was performed, in line with the Framework Method [35]. In accordance with this pragmatic approach, the analysis combined data from focus groups and individual interviews and involved 5 steps, performed mainly by the first author (EKK) in cooperation with the last author (GC). In the *first step*, all transcribed interviews were read to achieve familiarization and get an overview of the content. In the *second step*, an initial framework was constructed based on the different parts of the tool (ie, information and answering), which was then revised after the first interviews. In the *third step*, the transcribed data were indexed according to “codes” and sorted based on the initial framework. NVivo 11 software was used for data management. In the *fourth step,* the data extracts were reviewed together to ensure that similar content was sorted together and to determine whether the theme titles should be adapted. In the *fifth step*, data were summarized and displayed in a matrix in an MS Word file. Each subtheme was summarized based on the codes and raw data. The individual interviews, combined, were handled as one case and each focus group as separate cases. Data from each case was summarized separately before all cases were merged. The systematic data management using NVivo enabled easy access back to the initial subthemes and interview transcripts. Finally, the patient partner and coauthors read the summaries and were involved in refining the themes. Throughout the process, memos were written to summarize reflections, alternative interpretations, and potential amendments. Data collected from the web survey and self-reported characteristics were analyzed using descriptive statistics and are presented as numbers or medians and ranges. Analyses were performed using SPSS version 24 (IBM, Inc.).

### Ethical Approval

The study was approved by the Swedish Ethical Review Authority (no. 556-17 and 2020-03324).

## Results

### Design

The participatory design process grounded in user experiences resulted in a digital previsit tool. The following description of participants, the process for development, and user experiences provide insights into the rationale for amendments that were made.

### Participants

The study included a total of 22 individuals with stroke (9 women), with a median age of 59 (range 42-83) years, and a median of 51 (range 4-228) months since stroke onset. Together, the participants represented a wide range of individual characteristics (Table 1).

**Table 1:** Characteristics of participants included in the study.

Participants		Focus group 1 (n=3)	Focus group 2 (n=4)		Focus group 3 (n=5)	Interview, survey (n=10)^a^
Age, median (range)		67 (64-83)	65 (43-73)		55 (47-70)	54 (42-74)
Male sex, n		1	2		4	6
**Education (highest degree), n**						
	Mandatory	0		0	1	0
	High school	2		1	4	7
	University	1		3	0	3
**Source of income, n**						
	Work	0		0	1	6
	Sick leave	1		2	2	2
	Retirement	2		2	2	2
Months since stroke, median (range)		192 (168-228)	126 (72-156)		14 (4-24)	42 (13-144)
**Stroke characteristics (self-report), n**						
	Ischemic stroke	3		3	4	7
**Location**						
	Right	0		2	3	5
	Left	2		1	2	3
	Posterior	1		1	0	2
Communication difficulties (aphasia), n		2	1		2	5
Activities of daily living independency, n		2	4		4	8
Internet use daily, n		1	4		5	8

^a^One participant (male) answered the web survey but did not participate in an interview afterward.

### Development

The development process included 3 pilot versions of the tool (Figure 1) and stepwise alterations (Multimedia Appendix 2) before version 1.0 was completed (for visual presentation, see Multimedia Appendix 3).

In the conceptualization phase, the initial prototypes (webpages) were developed in collaboration with the first author and a service designer. During this stage, the focus was on user’s needs rather than fitting into a specific platform. The first digital pilot, version 1, included the following components: a logotype and title Strokehälsa [Strokehealth], introductory information, questions about health problems to be answered with yes or no, explanatory text (linked to “read more”) placed in direct connection with each question, and summary of results. The questions were in accordance with the PSC when applicable. The name Strokehälsa was chosen based on the aim of promoting health and improved life after stroke. The layout was intended to be clean and to avoid overwhelming text while remaining open to the possible addition of more information in the future. The information was layered using “read more” texts, with the aim of adapting the information level to each individual. The explanatory (read more) texts were inspired by existing patient information, such as pamphlets and booklets, as a starting point for gathering opinions.

**Figure 2:**
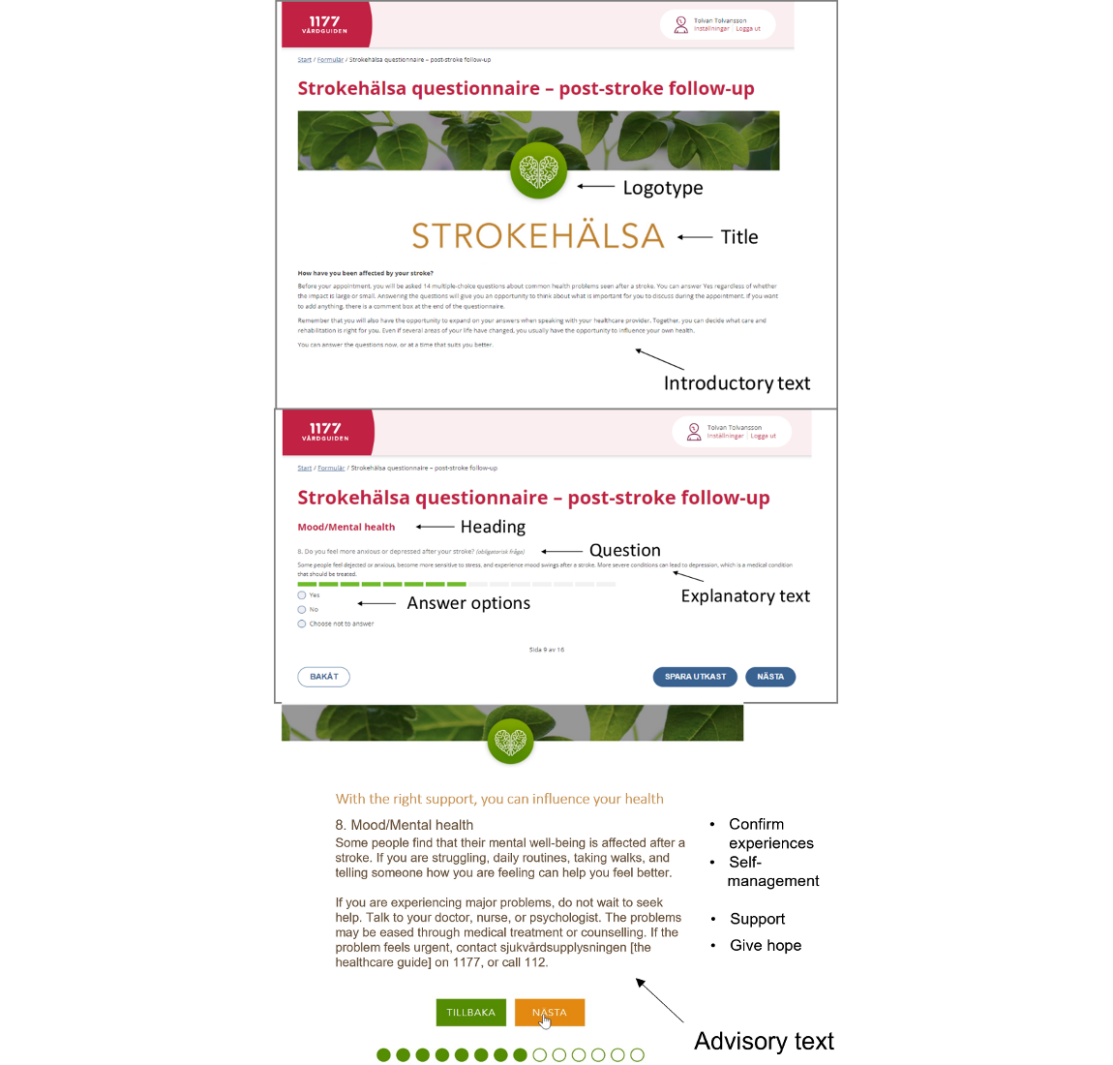
Screenshot with an overview of the core functions in the tool.

In the amendment phase, alterations were performed based on preliminary findings from the focus groups and the theoretical framework. The integration between the central components, validated questions, health information, and technical aspects was essential to improve usability. Thus, beyond revising the questions, adding real-life examples in the explanatory text enhanced usability. Important changes in pilot 2 were the inclusion of advisory texts, with brief information about support options and self-management, and a free textbox offering the opportunity to describe “other challenges.” In pilot 3, a general question about rehabilitation and a place for free-text comments were added. This pilot was incorporated into the national platform. However, the platform has some limitations regarding layout options, such as no hidden read more text option and predefined typography and colors. Figure 2 shows a screenshot of the patient view of the tool. The functionality of the platform includes sending the patient an email or SMS text message notification with an invitation to use the tool and to answer the questions before a care visit. Responsible health professionals can send version 1.0 of the tool to patients and view the summary of results (using a staff log-in at the secure platform 1177) before a care visit.

### User Needs and Experiences

Results regarding user experiences were based on the satisfaction survey and qualitative interviews.

#### Satisfaction Survey

**Figure 3:**
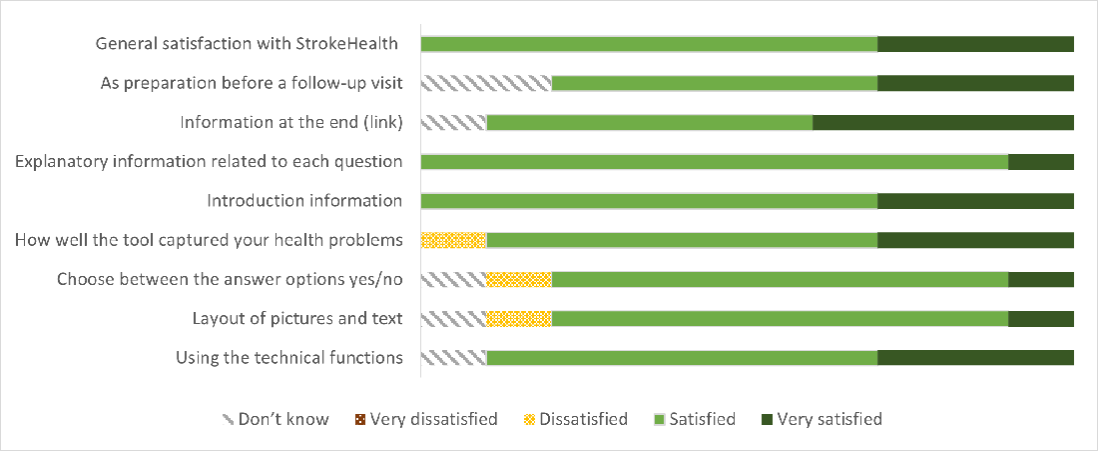
Satisfaction with using the tool (n=10).

Satisfaction with the tool was high (Figure 3). Most participants were satisfied, and all participants in the amendment phase would recommend use of the tool in clinical practice. Participants used different devices, with the majority using their mobile phone (n=16), followed by tablet (n=3), computer (n=2), and more than 1 device (n=1).

#### Themes Created Based on Focus Groups and Individual Interviews

##### Overview

**Figure 4:**
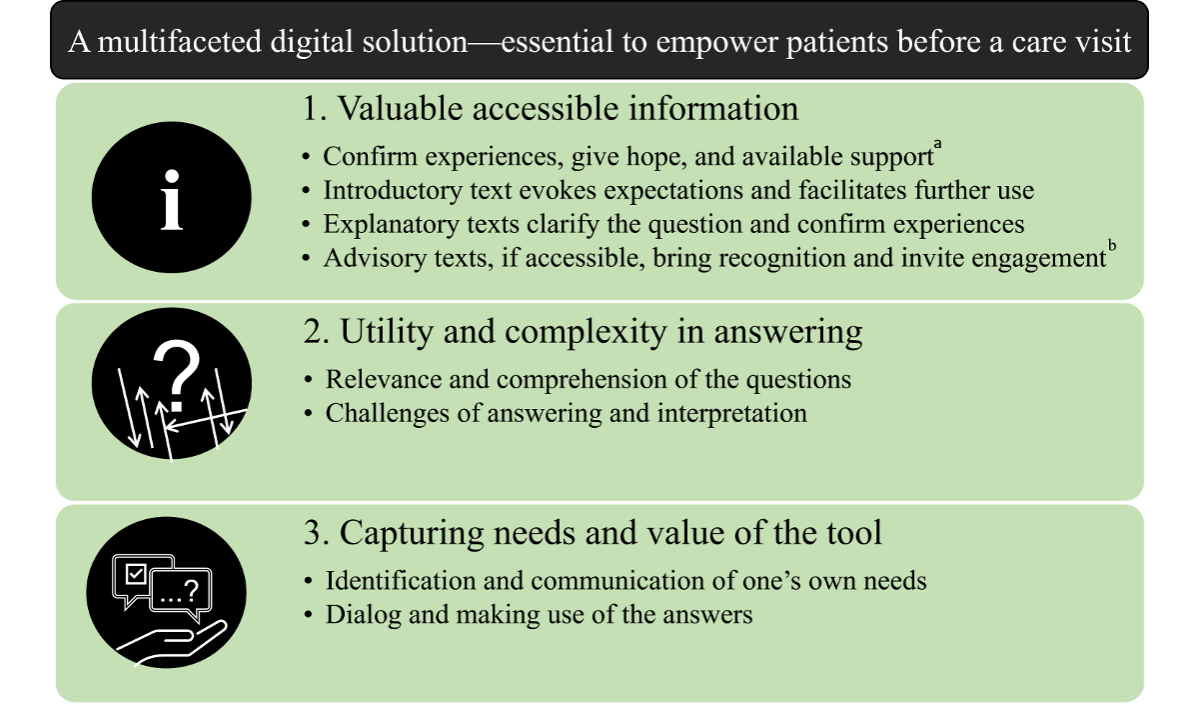
Themes and subthemes based on user experiences described in focus groups and individual interviews. a: Only data from focus groups; b: only data from individual interviews.

Analyses of focus group discussions and individual interviews were combined to clarify the meaning of the users’ experiences with the tool. Experiences were summarized in the overarching theme (*A multifaceted digital solution—essential to empower patients before a care visit*), main themes, subthemes (Figure 4), and quotes (Table 2).

**Table 2:** Quotes representing the themes in the qualitative analyses based on focus groups and individual interviews.

Themes and subthemes		Quotes
**Theme 1: Valuable accessible information**		
	Confirm experiences, give hope, and available support	P1^a^: *It’s like that* Moderator: *That you’re not alone - is that what you want to communicate/say?* P1: *Very important* P2: *Yep* Moderator: *(it’s) not just you?* P3: *It’s good to know you aren’t alone* [Focus group 1, people with communication difficulties]
	Introductory text evokes expectations and facilitates further use	*Yes, I expect that I can find out what I can do for myself to improve my health, as written now (introductory text), it provides an expectation but that wasn’t fulfilled. (Reads advisory texts). (…) Yeah yeah, but I get it, now that I’ve been able to read your link, why what is written there, is written there. *[P4, woman, 50 years old]
	Explanatory texts clarify the question and confirm experiences	*I think that the “read more” gave me enough information to be able to maybe change my answer to yes rather than no. *[P5, Focus group 2] *There was a little bit more (information) there and I think that it was easier to answer, like “yeah, that fits really well with the answer I want to give”, so I didn’t have to doubt (my answer). *[P6, man, 42 years old]
	Advisory texts, if accessible, bring recognition and invite engagement	*I don’t think I got it, no I didn’t get it clearly actually (....) I was like, kinda done with the questionnaire and so I was done.* [P7, woman, 53 years old] *Yes, I see it. Much easier to read (divided in sections and Strokehälsa layout) yes, it’s pretty fantastic really but, it’s actually the case, that I found it to be, that it was more interesting to read it that way. Because then you were a bit curious about what was on the next page, otherwise you see all the titles at once and then it’s, it looks like a lot, that this didn’t look like too much. *[P8, man, 66 years old]
**Theme 2: Utility and complexity in answering**		
	Relevance and comprehension of the questions	*I think that the questions were good, easy to answer, I didn’t think it was a problem for me to answer them. So, I didn’t see any obvious gaps, like “oh this, I didn’t get this or didn’t understand that.”* [P6, man, 42 years old]. P5: *Yeah, I never got into those questions if I answered no (Activities of daily living).* Moderator: *Do you think you miss out on people who don’t end up answering the follow up questions if they main questions are too broad?* P5: *That can happen.* P9: *Yes, I think so (too) because I just saw that if you click on yes there are more questions that come up.* P10: *And yeah, it’s easy to press no.* [Focus group 2]
	Challenge of answering and interpretation	*No, I think that some of the questions could have been so that you only had one choice, I told you a bunch of times that they’re on the edge, that if you could squeeze in a third option, so it’s yes or no, there’s something in between, you heard I was in a grey zone several times (between yes and no), you can maybe put it like that.* [P11, man, 59 years old] *It would be if you could add some kind of comment somewhere. Because if none of the questions are appropriate you could just write something yourself. But that’s usually the problem, that you don’t write anything yourself, just answer yes/no, but the opportunity to write something would be good. * [P12, man, 56 years old]
**Theme 3: Capturing needs and value of the tool**		
	Identification and communication of one’s own needs	P13: *It could be a support by prompting certain questions that you maybe hadn’t thought of* P14: *Or it prompts the questions that you’d thought of but had kind of just swept under the carpet. (...) Getting these questions and thoughts aired so that you can get your thing moving* P15: *You can get answers to questions you maybe didn’t understand or didn’t get answers to.* [Focus group 3] *If it’s there (in the tool), maybe you’ll be brave enough to bring it up (sex), with your doctor. Otherwise, it might be a bit too sensitive to mention it.* [P8, man, 66 years old]
	Dialog and making use of the answers	*No, it’s enough that you just answer with a yes, if that yes gets some attention at a doctor’s appointment.* [P7, Woman, 53 years old] *I’m not saying that I speak for everyone, but I think that lot of people want to have the possibility, at least, to tell someone how you feel, that meeting people in between is preferable.* [P16, man, 51 years old]

^a^P: participant.

##### Valuable Accessible Information

###### Overview

This theme comprises the perceived value of information to confirm experiences after a stroke episode and to facilitate further use of the tool.

###### Confirm Experiences, Give Hope, and Available Support

Participants emphasized their need for reliable and targeted information. Participants strongly agreed that they wanted information to confirm that their health problems were common, as well as unique, and related to individual prerequisites (eg, emotional reactions). Furthermore, participants emphasized the nature of information for showing people that they are not alone and for bringing hope. Those who had lived with their condition for several years offered suggestions to encourage people to do something fun, to exercise, to have goals that are important to them, and to not give up. They also mentioned the value of meeting others and the benefits of providing information about patient organizations.

The scope of information was discussed. Some users wanted a lot of information, including web links, access to video clips, and “GPS coordinates” to local services. Others emphasized that information must be brief and easy to read. Participants further identified a risk of disappointment if they found only answers to their questions without getting any solutions. Therefore, they suggested information designed to provide advice related to each question in the tool, for example, information about subsidized dental care and rehabilitation services.

###### Introductory Text Evokes Expectations and Facilitates Further Use

Overall, the introductory text was seen as concise, simple, and clearly stating the purpose. The layout was considered clean and appealing, with the heart-brain symbol and green leaves. After this positive first impression, participants recognized a gap between the expectations created by the introductory text and the content of the tool. One participant described that she had expected more from the tool about improving health based on the introductory text. Notably, she had not read the advisory texts, and changed her mind when she read these texts during the interview. Several participants came up with suggestions for how the text could be revised to fill this gap.

###### Explanatory Texts Clarify the Question and Confirm Experiences

In general, the explanatory texts (placed underneath each question) were considered important to facilitate answering and confirm the range of related issues. Participants said that the texts clarified the questions and helped them determine whether they had appropriately interpreted them. This perception was confirmed during the interview, when one participant and her next of kin were “thinking aloud” when answering. Although some people may need help, for example, because of communication difficulties, the scope of the text was perceived as adequate. Participants generally found it valuable to read the explanatory texts, as they confirmed experiences as common and reduced potential feelings of being atypical. In pilot 1, not all participants intuitively recognized the hidden read more texts. However, when they did read them, they perceived these texts as improving their ability to give an informed answer.

In the explanatory texts, the balance between general explanations and specific examples was recognized as important. Some participants wanted more examples, whereas others felt that a general description was better. One participant mentioned that she could pay her bills but still had cognitive difficulties that interfered with her performance, for example, she was easily disturbed. Another issue mentioned was an inconsistency between the question and the provided examples. This inconsistency could be misleading for those who felt that they sufficiently managed specific activities, for example, transfer to a car, but experienced difficulties in situations demanding caution. Suggestions to improve the texts were highlighted, for example, relating to “walk and move,” “pain,” and “fatigue.”

###### Advisory Texts, If Accessible, Bring Recognition and Invite Engagement

Participants appreciated the tonality of the advisory texts, embracing encouragement to engage in their own care and rehabilitation. The scope and content of the information were considered optimal. Although some participants had previously been provided similar information, one said that seeing the information made him realize that the support he had received was adequate. They suggested naming selected support associations to enhance easy access. Despite comments that the texts were beneficial, participants also described obstacles related to accessibility. Several participants did not notice the advisory text when it was presented after submitting their answers, as they had a feeling of being “done.” It was suggested that it would be helpful to include an introduction encouraging people to read the advisory text. Participants also recommended rewording the title to include what “you can do yourself” and “how you can get support.” Suggestions to improve accessibility included placing a web link before answering or in more direct connection to each question. Additionally, the text layout was perceived as important. Those who saw all text in black on one page and compared it with the link to a text divided into sections with the specific “Strokehälsa layout” preferred the latter.

##### Utility and Complexity of Answering

###### Overview

This theme comprises the complexity of answering and includes perceptions of the questions, answer options, and interpretation of answers.

###### Relevance and Comprehension of the Questions

Overall, the health problems included were considered relevant and to encompass a broad range of topics. However, it was reported that some areas could be missed, for example, fatigue, vision, and swallowing. Some felt that the number of questions was just right, but others thought of additional questions to include. Answering questions not relevant to oneself was not considered a problem. In general, the questions were considered easy to comprehend. However, many participants identified the risk that if a person answered “no” to the overall question regarding “activities of daily living,” they would miss the follow-up questions, as constructed in pilot 1. Participants expressed concerns that some headings were hard to understand, particularly secondary prevention. By contrast, others reported that headings, such as cognition and spasticity, contributed to their learning.

###### Challenges in Answering and Interpretation

Participants discussed potential answer alternatives, such as number of options, grading, and the use of free text. For most participants, answering yes or no in combination with the related explanatory text worked out well. However, participants still expressed their wish to explain their answers—for example, that “yes” means that they are slower to do things. Participants described experiences of frustration when answering, particularly when they only experienced minor health problems. One woman described frustration when she answered “yes” to “Activities of daily living,” but only meant that she had trouble tying her shoelaces. Some argued that multiple answer options would make it easier. By contrast, others saw a risk of complicating things, especially participants with communication difficulties.

Proposals to use free text rather than yes/no were met with counterarguments. Participants who had unsatisfactory experiences using yes and no options said that a combined solution would have improved answering. In later pilot versions, when “other concerns” and free-text boxes were added, participants valued the opportunity to raise additional issues and provide individual comments. Apart from the potential limitations of yes and no, participants acknowledged that these options made answering quick and easy. Other suggestions included using a consistent approach to subsequent questions, and the possibility of having the questions read with sound. Participants thought that the pilot versions incorporated into the platform generally functioned well. However, some failed to submit or thought that it was not easy enough to change their answers, and thus offered suggestions to improve navigability.

Interpretation of the answers was discussed. Some participants were concerned that yes and no options may not provide sufficient information to health professionals. They also felt that the phrase “more difficult after your stroke” could be challenging when they experienced a health problem as more problematic but not more difficult. In particular, participants with minor impairments reported feeling that they did not want to exaggerate the problem. They further described that their abilities were likely to shift over time or to be situation dependent, such that the answers were not unambiguous. Next of kin highlighted that the user’s perception can be opposite to that of his/her relative, indicating different views of the situation, especially several years after the stroke. The tool was viewed as a rough measure.

##### Capturing Needs and Value of the Tool

###### Overview

This theme highlights the tool’s potential to identify health problems, and the importance of dialog with a health care professional at the care visit.

###### Identification and Communication of One’s Own Needs

Most participants expressed that having access to the information and identifying health problems through the tool would have supported them in communicating their needs. Increased knowledge about available support was described as essential for being able to act and seek help. One participant said it would enable people to drive their case forward. Participants recognized that they often forget to bring up issues and acknowledged the benefits of making one’s health problems visible and easier to explain. Some described that insight into health problems as part of the disease picture would have encouraged them to ask the health professional questions, for example, regarding incontinence and sexuality. By contrast, one participant reflected that the absence of health problems in the tool could lead to a patient not associating this problem with his/her condition, and thus to be less likely to discuss it. Some described experiences of facing new problems after some time, for example, fatigue and return to work, and regarded the tool as helpful in this context. People with communication difficulties and 1 next of kin thought that a care visit could be enhanced by using the tool beforehand. Nevertheless, it was recognized that not all people can use the tool as an aid to identify their needs and that some would prefer a paper version.

###### Dialog and Making Use of the Answers

Participants strongly emphasized that use of the tool should be followed by a dialog between the health care professional and the patient. Many described a desire to explain what they meant by their answers. One participant expressed that it was sufficient to answer “yes” in the tool if the “yes” can be elaborated on in a subsequent conversation at the care visit. Another participant felt that meeting with people was preferable, compared with free text or ticking a box; however, he could see value of the combination of both. Some suggested that the use of their answers at the care visit was a fundamental prerequisite for the usefulness of the tool. Although participants considered the answers useful for health care professionals, there were concerns regarding whether they would have the necessary staff resources to fully implement this new digital service and change their ordinary routines.

## Discussion

### Principal Findings and Comparison With Prior Work

A participatory co-design was used to develop a digital previsit health care tool based on experiences of people with stroke, health professionals, and researchers. Integration of health information, validated questions, and digital functionality contributed to the development of a tool perceived as easy to use. The findings suggest that a condition-specific tool can confirm commonly perceived experiences and give targeted support and that the elements in the tool can be adapted to other health conditions.

This study included discussions about the optimal scope of information and questions in the tool. The theme *Valuable accessible information* describes needs that can potentially be met by eHealth services [13]. Besides condition-specific information, participants in this study particularly valued information encouraging them to be involved in their care. Accordingly, the information in the tool was created with the goals of preparing patients for active participation and enhancing their involvement in decision making [1]. Nonetheless, given the various levels of eHealth literacy [18] and the different expectations among participants, it was challenging to provide information at an optimal level for the group.

During the conceptualization phase, participants valued reliable information of various kinds, and the amount of information to be included was not yet determined. Of note, on the secure platform, the possibility of including and layering a larger amount of information was restricted, so that only brief information was included. Furthermore, the validated questions in the tool were perceived to cover the most important topics. These findings reinforce those of a previous study suggesting that the PSC directly or indirectly covers most problems [36]. The risk of health problems (eg, nutrition, sex life, and fatigue) not being covered has been previously discussed [31,37] and was mentioned in this study. A previously reported digital tool developed for long-term conditions included a more comprehensive list of nonvalidated items [17], but its usability in a clinical context remains to be tested.

It is important to reflect on whether adding more questions or measurements counteracts the perceived usability of a tool [24]. When an adapted version of the PSC was employed in combination with other measurements in a digital platform, it was used by only 11.8% of the patients, although they were offered training to use it [26]. However, usability aspects are not provided in detail. In our study, instead of increasing the number of questions, the principal decision was made to adjust the texts and encourage users to use the separate free-text option at the end, when appropriate. The hope was that the design of the tool and mode of information provision would accommodate a large group of people with long-term conditions, among whom eHealth literacy can be low [18]. Evaluation in clinical practice is important to explore whether the scope and level are optimal. Nevertheless, the level of information and questions in version 1.0 were considered a good starting point for empowering patients to be actively involved in their conversations with health care professionals.

Within the theme *Utility and complexity of answering*, aspects of reporting perceived needs and health problems were highlighted. Previous findings indicate that identifying unmet needs through self-report is complex for people [38]. Unmet needs are influenced by perceptions and experiences, such as value of independence or insights regarding available services. Participants in this study stated that their abilities were likely to shift over time and because of changing circumstances, which is consistent with previous research [38]. Moreover, people may not be capable of fully understanding and answering the questions in relation to their own situation [9,18].

In this study, a combined solution was used to facilitate answering for a broad range of patients. First, it was considered best to provide the answer options of yes/no/“choose not to answer” together with a free-text option at the end. People seem to take longer to consider the information in a question when a yes/no format is used compared with ticking a box in a list [39]. This format encourages people to think about the question in relation to their own situation, thereby preparing them for shared decision making [1]. Second, the explanatory texts underneath each question were revised as an additional solution. When using the questions at a care visit, the related dialog has been highlighted as important for ensuring that health problems are identified [31]. Similarly, participants described the explanatory texts as an asset when answering, although they did not consider the texts to replace the dialog at a visit. Third, answering was improved by clarifying that patients could explain their answers at the care visit. Overall, it is likely that the complexity of answering was decreased through the combined solutions, including the questions, answer alternatives, and the information in the tool.

Throughout the development process, multifaceted solutions were applied to accommodate limitations revealed in the interviews and to improve usability. The view of shared decision making as a holistic process, including visit preparation and the visit itself, is congruent with the service design approach [19]. Solutions to complex problems can be better solved holistically; for example, a digital tool used as part of a service [20], compared with just fragmented text presented out of its context.

In this study, the true value of the tool was perceived to depend on whether identified health problems would be addressed in the conversation at the care visit. Self-reported measures completed beforehand and received by the provider lead to patients more commonly discussing nonspecific long-term health problems, without prolonging the care visit [40]. However, using checklists [41] and previsit tools alone may not result in benefits for patients [42]. Several components are important for the delivery of effective care, such as infrastructure, people resources [43], and health care professionals’ skills and motivation to provide a person-centered conversation [1,41]. Moreover, successful implementation requires consideration of the meaning value for all users, and how the team can use the tool to change their routines and improve services [44]. However, participants in this study suggested that a tool that included information could empower people to act on their own more readily and seek support.

In our study, the users’ needs were in focus, in line with the service design procedure [20], and in contrast to being restricted by an existing digital system. In recent years, digital maturity in the population has increased [3], and digital health systems have changed dramatically. Therefore, attention has focused on developing a flexible and sustainable solution [28]. Only later in the development process were the core functions in the pilots transferred to a health platform that both patients and providers know and trust. The intention was for the tool to be easy to copy, modify, and connect to other platforms and contexts. Usability for the individual was considered high, as people could go through the tool quickly and found it appropriate in relation to their needs. This easy access means that the tool is more likely to be used [20,24]. Nevertheless, to accommodate patients with low eHealth literacy [10,18], a paper-based version in various languages will be developed. Together with guidelines suggesting digitally based information and support [7], the tool Strokehälsa could contribute to a move toward a more proactive health care team and patient preparedness [4]. Participants’ responses supported the value of the tool and its potential to capture their needs, but both need to be tested in a larger sample in a clinical setting.

### Limitations

A strength of this study is the comprehensive participatory approach, including mixed methods, enabling a deep understanding of user experiences. However, some limitations must be addressed. First, despite purposive sampling, selection bias cannot be excluded. Most participants were independent in activities of daily living and used the internet daily. Furthermore, because of COVID-19, people had to connect to the tool digitally on their own device and participate in a phone interview, which may have limited the recruitment of people less familiar with digital tools in the amendment phase. However, support from next of kin was allowed, and the early focus groups were conducted face to face. Of note, the remote data collection worked out better than expected and yielded rich data. Altogether, the use of several sources allowed triangulation of data and a broad range of participants from different settings. The detailed descriptions of participant data analysis strengthen the transferability of the findings to other contexts.

Second, in line with the qualitative approach, attempts were made to sustain rigor and reflexivity, and interpretations were influenced by prior knowledge, for example, about stroke, the PSC, and person-centered care. The members of the research team were part of the co-design process, emphasizing the collective creativity with all stakeholders [23]. Although involvement of the researcher is part of the method, it cannot be excluded that a researcher role could have affected participant statements. However, they were encouraged to speak freely and contribute to improvements in the tool. Suggestions from participants, the expert panel, and the research team were systematically registered along with reflections in memos [35]. If controversies arose during decisions, advice was sought from members of the expert panel. Third, from a co-design perspective, the level of partnership in the study can be discussed. The patient partner was not directly involved in the initial workshop built on previous research. Although interaction on equal terms is the goal, it may not be realistic or possible for the same individual to be involved in all stages, for example, because of cognitive impairment or fatigue [29]. Nevertheless, the patient partner was continuously involved in the co-design and research process. Additionally, feedback was obtained from people with long-term conditions, health professionals providing care, and researchers in different fields. The participatory approach through service design principles led to the creation of a tool based on user needs (updated versions of the tool can be found on a webpage [45]).

### Conclusions

The development process with a participatory approach resulted in a previsit digital health care tool viewed as useful for people with stroke. In this process, the integration of health information, validated questions, and digital functionality was essential to overcoming the complexity of responding to the tool’s questions. Even when questions were perceived as easy to comprehend, the additional information supported answering and confirmed patients’ experiences. Moreover, the information encouraged people to develop their answers in dialog with the health care professional. However, larger studies that include evaluation in conjunction with a clinical visit are needed. The tool is freely shared to be adapted and improved in different contexts for ecological validity.

## Appendix

Multimedia Appendix 1 The question guide used in focus groups and individual interviews. (a: Only in the focus groups; b: Only in the individual interviews).

Multimedia Appendix 2 Description of amendments and revisions in the pilot versions. a: PSC= post-stroke checklist, b: ADL= activities of daily living.

Multimedia Appendix 3 Screenshots of the first prototype and version 1.0 of the digital tool Strokehälsa.

## Abbreviations

PSC: Post-Stroke Checklist
Strokehälsa: Strokehealth
TAM: Technological Acceptance Model
