# Supplementary material for: Development of a Digital Tool for People With a Long-Term Condition Using Stroke as a Case Example: Participatory Design Approach
Source: JMIR Hum Factors. 2022 Jun 3;9(2):e35478. doi: 10.2196/35478 (PMC9206198; doi:10.2196/35478)
Supplement: Multimedia Appendix 1 [file humanfactors_v9i2e35478_app1.docx]

**Multimedia Appendix 1.** The question guide used in focus groups and individual interviews. (^a^Only in the focus groups. ^b^ Only in the individual interviews).

| **Open ended questions** |
| --- |
| Thinking about using the tool -what are your spontaneous thoughts? |
| In what way would a tool like this aid as a support? How about in terms of use before a care visit?^a^ |
|  |
| How do you find the tool itself, the technical functions? |
| Thinking about the layout, what is your experience? |
| Regarding the introductory information, what is your experience? |
|  |
| Tell me about your experience when answering to the questions. |
| Thinking about the explanatory texts, what was your experience? |
| What kind of information do you wish to be included?^a^ |
| What kind of information do you believe could support improved health and quality of life after stroke?^a^ |
| Thinking of the information at the end (advisory texts), what was your experience? |
|  |
| What potential benefits can you identify if implementing this tool?^a^ |
| What potential barriers or risks can you identify if implementing this tool?^a^ |
| If changing something in the tool, what would you change? Anything missing? |
| What advice would you like to give us in the following work with the tool? |
| If you would recommend the tool to someone, what would you say?^a^ |
