# Supplementary material for: Development of a Digital Tool for People With a Long-Term Condition Using Stroke as a Case Example: Participatory Design Approach
Source: JMIR Hum Factors. 2022 Jun 3;9(2):e35478. doi: 10.2196/35478 (PMC9206198; doi:10.2196/35478)
Supplement: Multimedia Appendix 2 [file humanfactors_v9i2e35478_app2.docx]

**Multimedia Appendix 2.** Description of amendments and revisions in the pilot versions. ^a^PSC= post-stroke checklist, ^b^ADL= activities of daily living.

| **Conceptualisation phase:** Pilot #1, used in the focus groups. | **Amendment phase:** Pilot #2, used in the first four interviews and Pilot #3, used from the fifth interview. | **Amendment phase:** Version 1.0, ready to test with patients. |
| --- | --- | --- |
| **Design and platform** |  |  |
| Website constructed without regulatory constraints. | Standards as in the national health platform (1177), log in was not required. | Integrated in the regulated health platform 1177 demanding secure log in. |
| Typo, colours and logo with green leaves and heart/brain symbol trough out the tool. | 2# Logo with green leaves and heart/brain at first page. Other functions and format were predetermined in the platform.  3# External link was designed according to design principals in 1#. |  |
| **PSC questions^a^** |  |  |
| 1#PSC Q1 Heading “secondary prevention” and question “*Have you received* advice about health related……” | 2# Heading “Prevent another stroke” | Q1“*Do you want* information about how you can prevent another stroke” |
| 1# PSC Q3. Heading mobility and question“..are you finding it more difficult to walk or move safely from bed to chair”.  Q3 Sub-question, ongoing rehabilitation | 2# Heading “walk and move”. Q3 “…from bed to chair” moved to explanatory text.  3# Sub question was removed and replaced with a general question about ongoing rehabilitation at the end of SH. |  |
| Since your stroke was placed above each question as “Please answer since your stroke..” The text “or last exam” was removed. |  | “Since your stroke” was moved to the end of each question |
| **Answer alternatives** |  |  |
| Yes/no for first order questions |  | Additional alternative, “choose not to answer” |
| Several sub questions (ADL^b^) with “Tick boxes” at the same page. | 2# Consequently, one question on each page with yes/no. | Additional alternative, ”choose not to answer” |
| 1# Only the original PSC areas were included. | 2# A new area was added Q12“Other challenges” and response on who´s answered SH.  3# Added space for additional comments and question regarding ongoing rehabilitation. | Additional areas were added: Eat and drink and oral health. |
| **Introduction** |  |  |
| 1# Information about total number of questions, help to navigate afterwards and support the dialogue with staff. | 2# Changed wording regarding about the information-link.  3# Amendments including information at the end and possibility to make further explanations at the visit. | Minor amendments. |
| **Explanatory information** |  |  |
| 1# Hidden text with ”read-more” option. | 2# Visible text underneath the question. |  |
| 1# Short text to give an idea of possible content, not fully developed. Q11: removed relatives’ experiences. Removed any recommended actions. | 2# Further development to clarify the question and confirm experiences.  3# Amendments e.g., Q10 added intimacy and sex and adjustment in cognition. | Further amendments of wording and balancing general descriptions with specific examples e.g., fatigue mentioned in Q10. |
| **Advisory text** |  |  |
| Heading. 1# No content. | 2# “To think about before your visit”  3# to “Improve your health after stroke” | Heading “Do on your own and get support” |
| Main text. 1# No content. | 2# A one-sided page with introductory text, text related to each problem area followed by concluding text.  3# An external link with more “healty” layout divided in different pages. |  |
