# Supplementary material for: Development of a Digital Tool for People With a Long-Term Condition Using Stroke as a Case Example: Participatory Design Approach
Source: JMIR Hum Factors. 2022 Jun 3;9(2):e35478. doi: 10.2196/35478 (PMC9206198; doi:10.2196/35478)
Supplement: Multimedia Appendix 3 [file humanfactors_v9i2e35478_app3.pdf]

Visual description of initial prototyping, pilotversion 1 and Strokehälsa™ Version 1.0

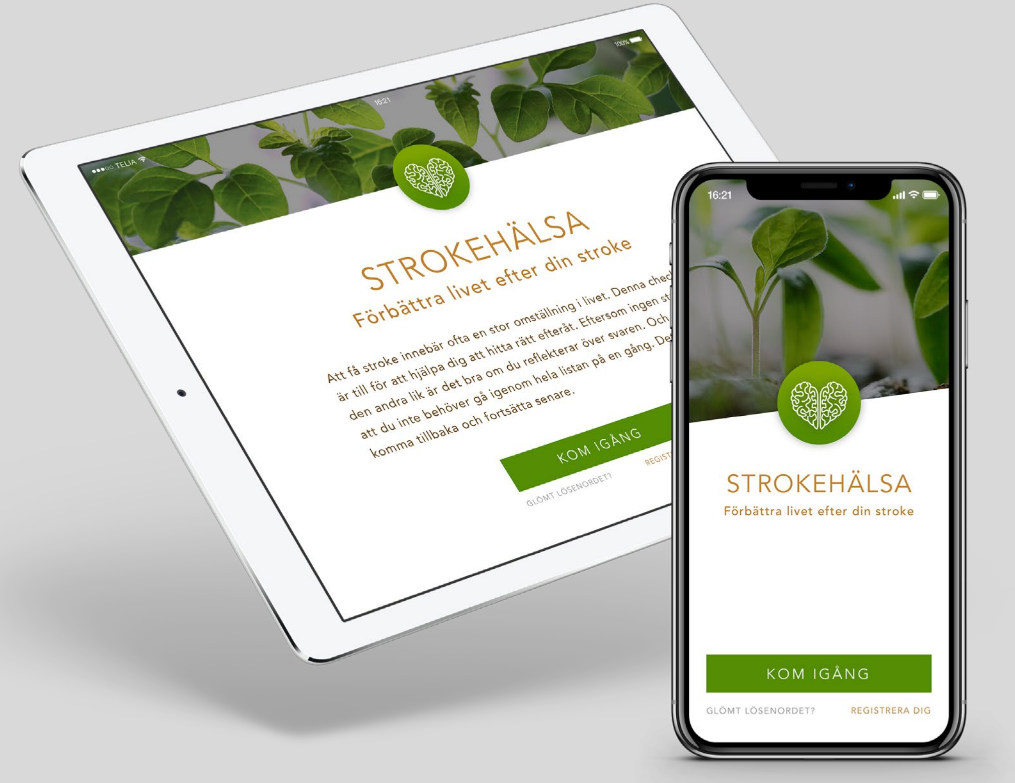

Initial prototyping

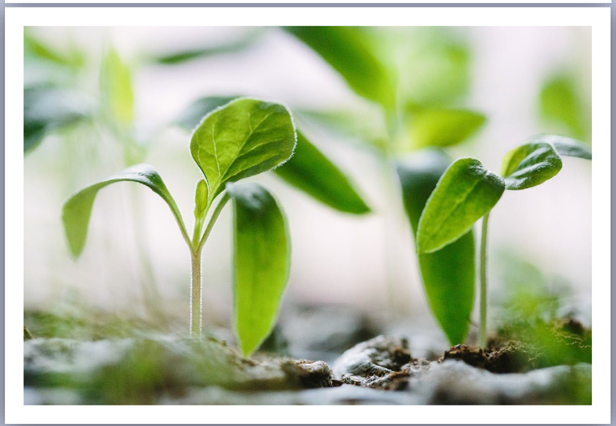

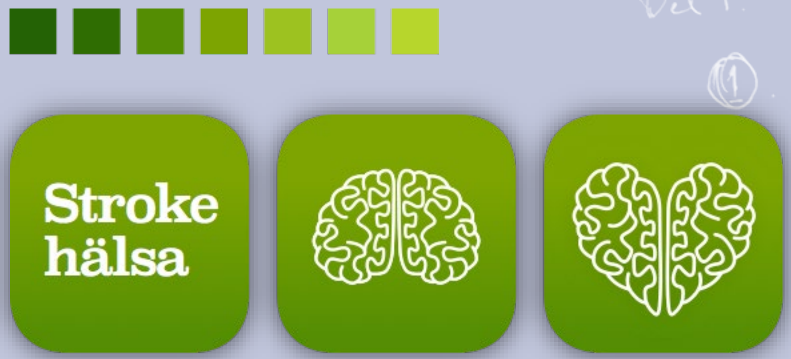

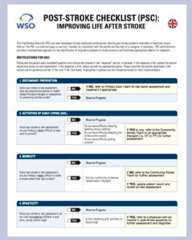

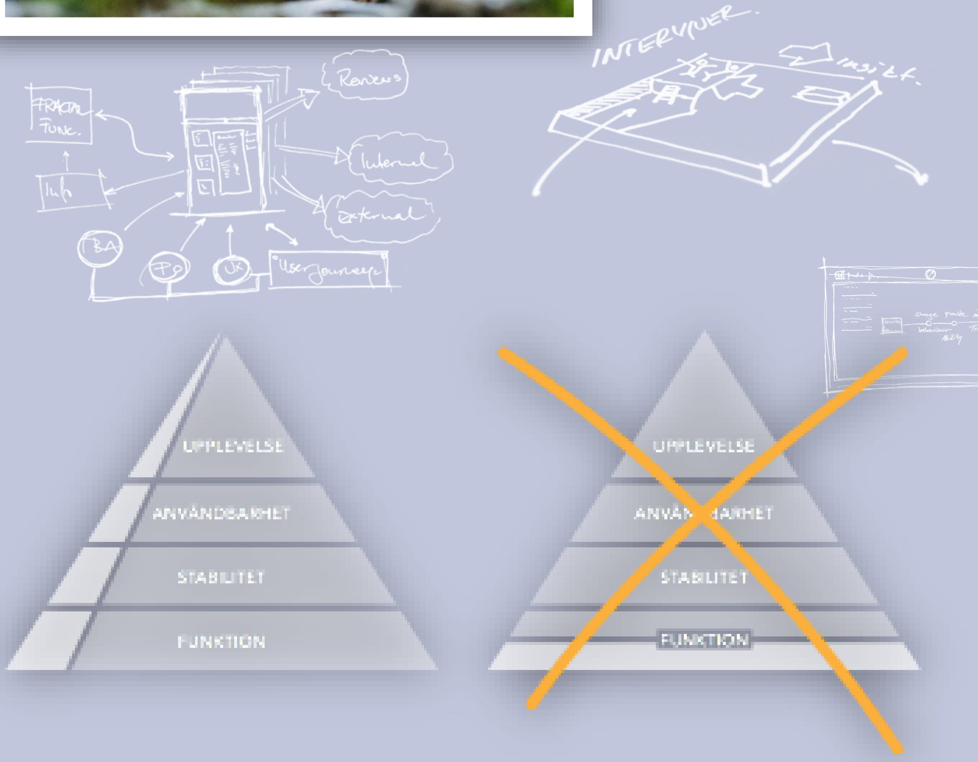

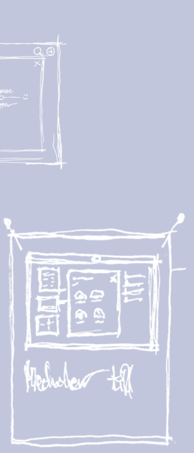

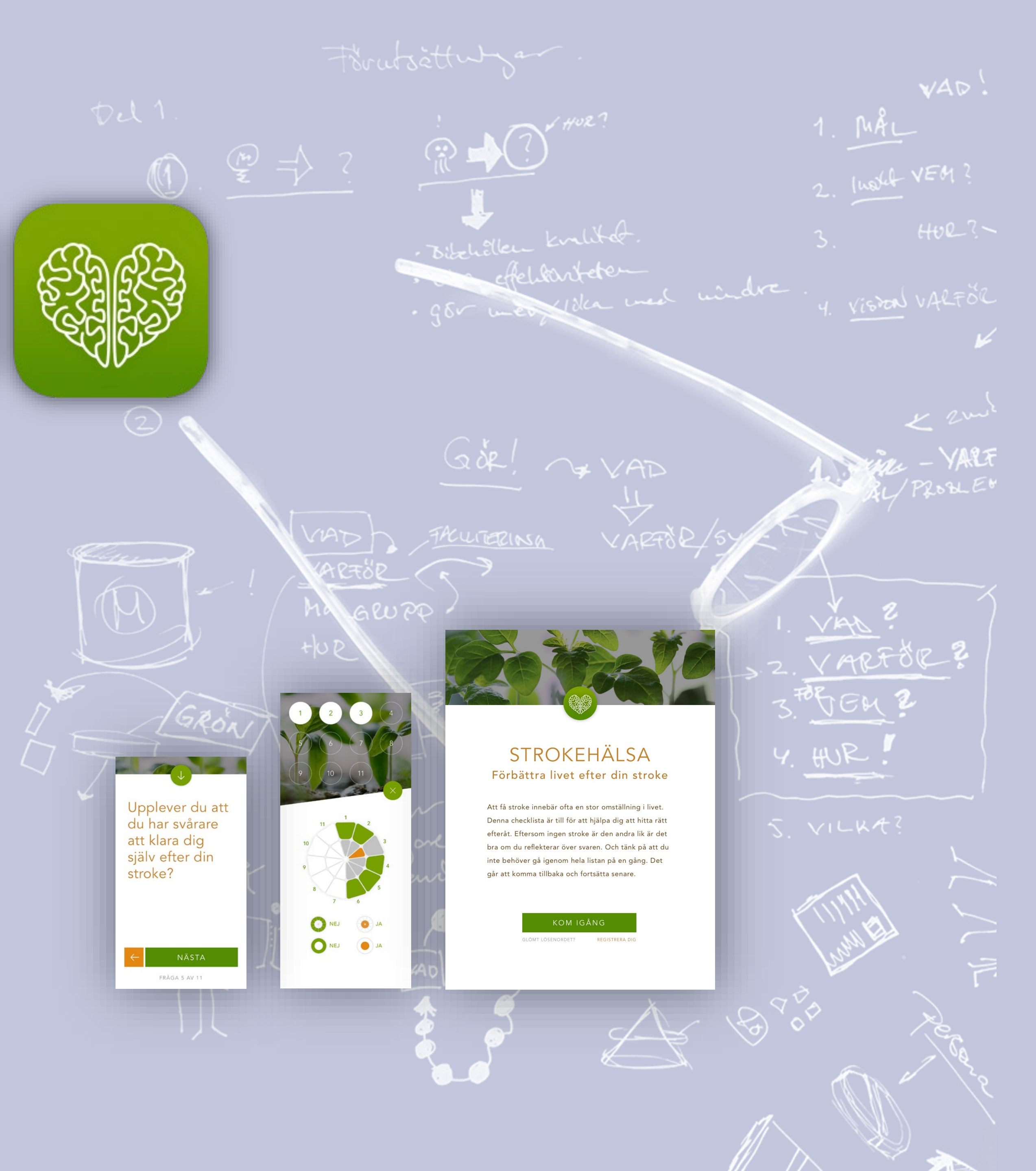

Patient-journey-mapping

Multi-professional support and self-management

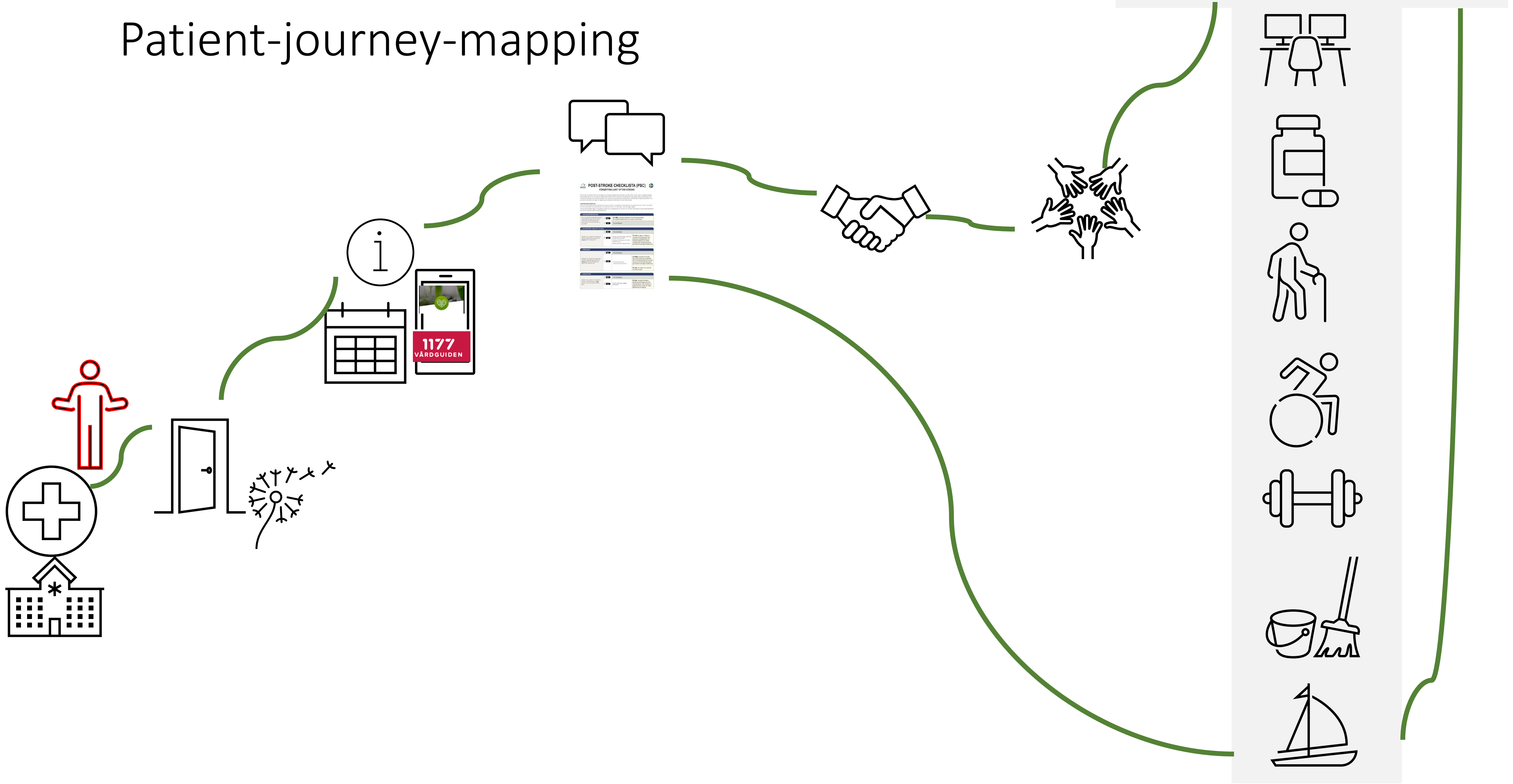

Personas

Examples of personas created to represent the potential user group.

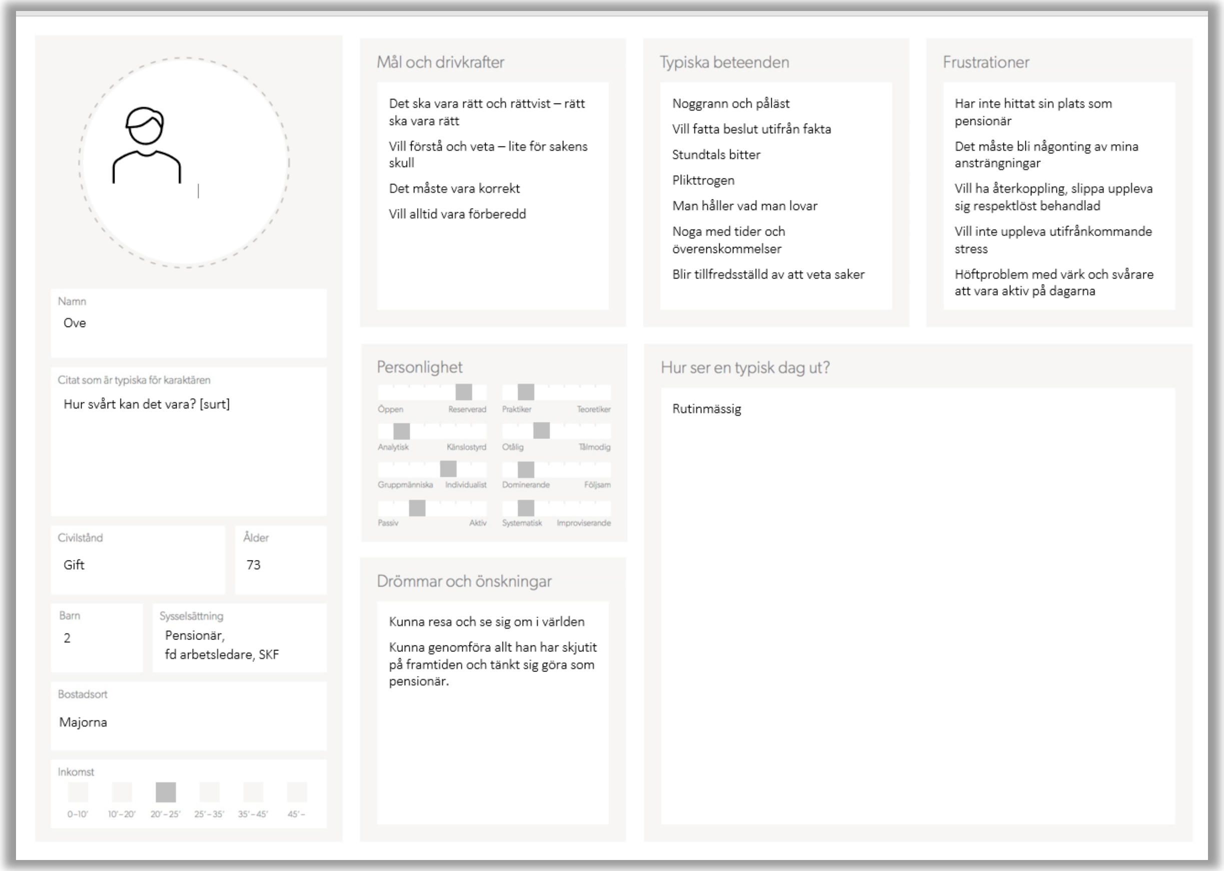

Mål och drivkrafter

Det ska vara rätt och rättvist – rätt ska vara rätt

Vill förstå och veta – lite för sakens skull

Det måste vara korrekt

Vill alltid vara förberedd

Typiska beteenden

Noggrann och påläst

Vill fatta beslut utifrån fakta

Stundtals bitter

Plikttrogen

Man håller vad man lovar

Noga med tider och överenskommelser

Blir tillfredsställd av att veta saker

Frustrationer

Har inte hittat sin plats som pensionär

Det måste bli någonting av mina ansträngningar

Vill ha återkoppling, slippa uppleva sig respektlöst behandlad

Vill inte uppleva utifrånkommande stress

Höftproblem med värk och svårare att vara aktiv på dagarna

Namn

Ove

Citat som är typiska för karaktären

Hur svårt kan det vara? [surt]

Civilstånd

Gift

Ålder

73

Barn

2

Sysselsättning

Pensionär,
fd arbetsledare, SKF

Bostadsort

Majorna

Inkomst

0–10' 10'–20' 20'–25' 25'–35' 35'–45' 45'–

Personlighet

Öppen Reserverad Praktiker Teoretiker

Analytisk Känslostyrd Otålig Tålmodig

Gruppmänniska Individualist Dominerande Följsam

Passiv Aktiv Systematisk Improviserande

Drömmar och önskningar

Kunna resa och se sig om i världen

Kunna genomföra allt han har skjutit på framtiden och tänkt sig göra som pensionär.

Hur ser en typisk dag ut?

Rutinmässig

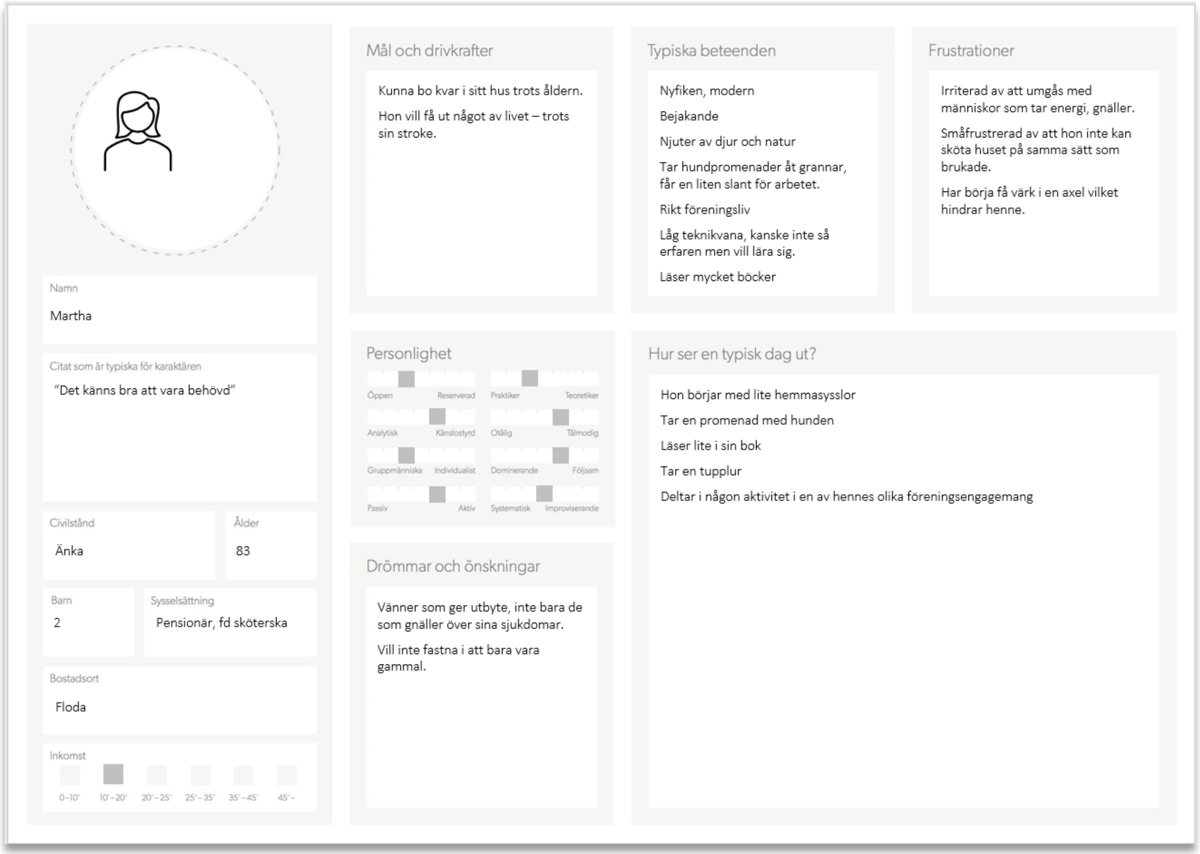

Mål och drivkrafter

Kunna bo kvar i sitt hus trots åldern.

Hon vill få ut något av livet – trots sin stroke.

Typiska beteenden

Nyfiken, modern

Bejakande

Njuter av djur och natur

Tar hundpromenader åt grannar, får en liten slant för arbetet.

Rikt föreningsliv

Låg teknikvana, kanske inte så erfaren men vill lära sig.

Läser mycket böcker

Frustrationer

Irriterad av att umgås med människor som tar energi, gnäller.

Småfrustrerad av att hon inte kan sköta huset på samma sätt som brukade.

Har börja få värk i en axel vilket hindrar henne.

Namn

Martha

Citat som är typiska för karaktären

"Det känns bra att vara behövd"

Civilstånd

Änka

Ålder

83

Barn

2

Sysselsättning

Pensionär, fd sköterska

Bostadsort

Floda

Inkomst

0–10' 10'–20' 20'–25' 25'–35' 35'–45' 45'–

Personlighet

Öppen Reserverad Praktiker Teoretiker

Analytisk Känslostyrd Otålig Tålmodig

Gruppmänniska Individualist Dominerande Följsam

Passiv Aktiv Systematisk Improviserande

Drömmar och önskningar

Vänner som ger utbyte, inte bara de som gnäller över sina sjukdomar.

Vill inte fastna i att bara vara gammal.

Hur ser en typisk dag ut?

Hon börjar med lite hemmasysslor

Tar en promenad med hunden

Läser lite i sin bok

Tar en tupplur

Deltar i någon aktivitet i en av hennes olika föreningsengagemang

Pilot version 1

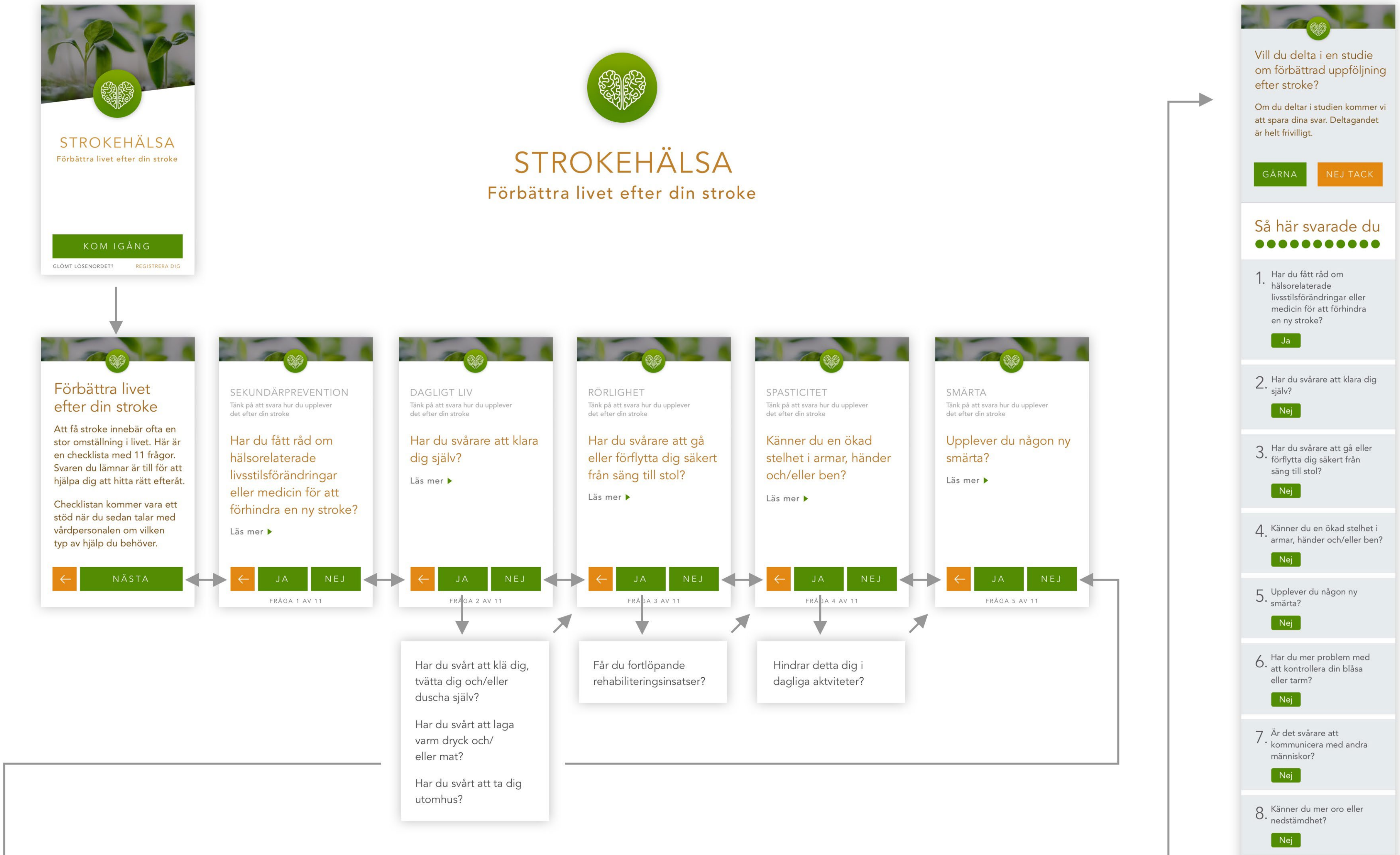

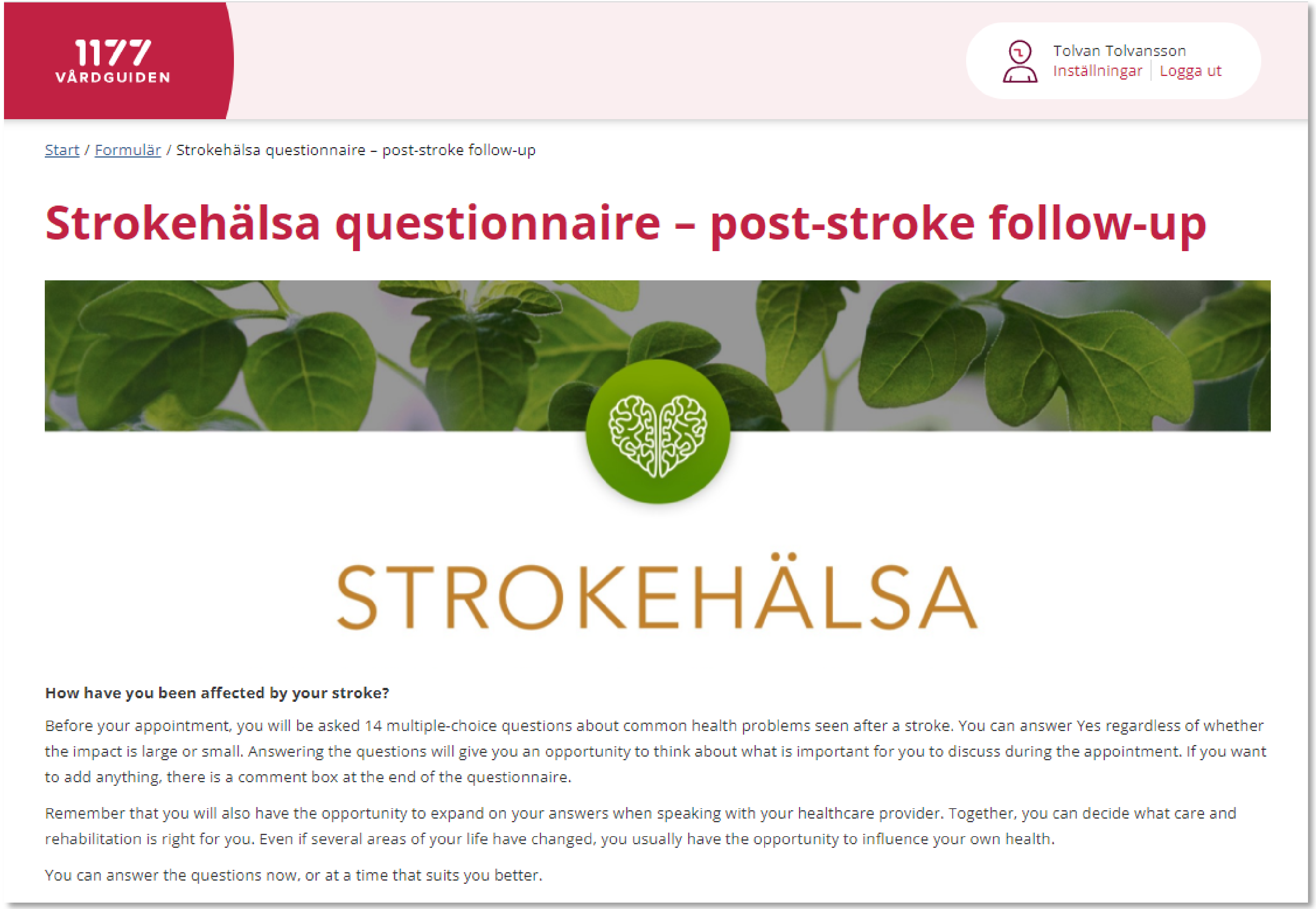

Strokehälsa™

Version 1.0

The following presentation includes screenshots of the version 1.0, incorporated in a secure platform Health care guide 1177.

The latest updated version of Strokehälsa is available at: www.gu.se/neurovetenskap-fysiologi/strokehalsa

#This first slide is based on a revised, version 1.1 including English text.

Introductory text

1177 VÅRDGUIDEN

Tolvan Tolvansson
Inställningar | Logga ut

Start / Formulär / Strokehälsa - uppföljning efter stroke

Strokehälsa - uppföljning efter stroke

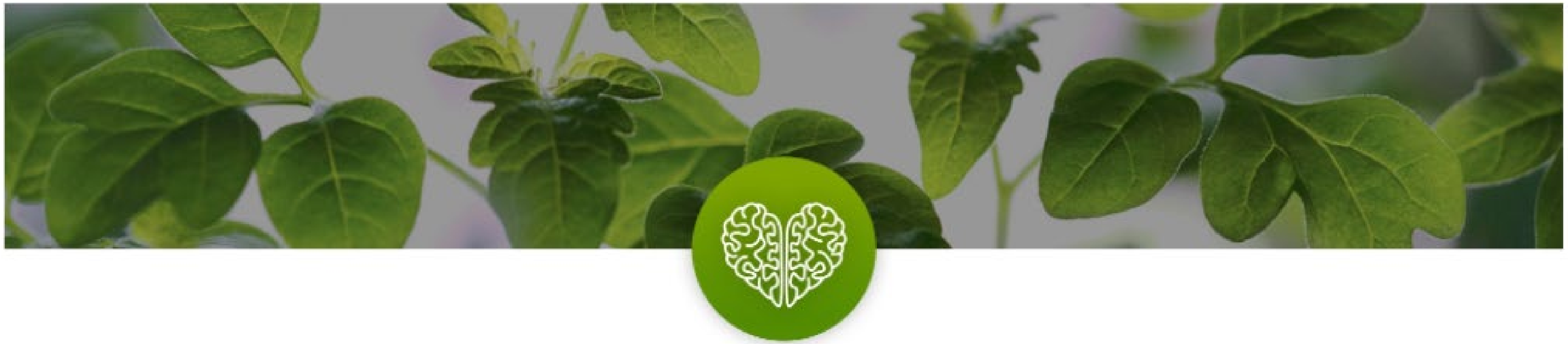

STROKEHÄLSA

Förbättra livet efter din stroke

Hur har du påverkats efter din stroke? Mycket, lite eller inte alls?

Formuläret *Strokehälsa - uppföljning efter din stroke* består av två delar. Först får du svara på 14 frågor och skicka in dina svar. Därefter kan du läsa mer om vad du kan göra själv och hur du kan få stöd. Genom att du delar med dig av dina svar kan du sedan lättare föra en dialog med vårdgivaren. Vid ditt personliga besök har du möjlighet att utveckla dina svar.

Tillsammans kan ni komma fram till vilken vård och rehabilitering som passar dig. Även om flera områden i livet förändrats har du oftast möjlighet att själv påverka din hälsa.

Questions

1177 VÅRDGUIDEN

Tolvan Tolvansson
Inställningar | Logga ut

Strokehälsa - uppföljning efter stroke

Förhindra ny stroke

1. Vill du få råd om vad du kan göra för att förhindra en ny stroke? *(obligatorisk fråga)*

Risken för en ny stroke kan minskas betydligt genom en kombination av livsstilsförändringar, medicinering och blodtryckskontroller. Livsstilsförändringar kan vara regelbunden motion, god kosthållning, att sluta röka och viktnedgång.

- ○ Ja
- ○ Nej
- ○ Avstår från att svara

BAKÅT SPARA UTKAST NÄSTA

Strokehälsa - uppföljning efter stroke

Vardagliga aktiviteter

2. Har du svårare att klara dig själv i vardagliga aktiviteter efter din stroke? *(obligatorisk fråga)*

Här menas vardagliga aktiviteter som att duscha, klä på sig, laga mat eller att kunna ta sig ut.

- Ja
- Nej
- Avstår från att svara

Sida 3 av 16

BAKÅT SPARA UTKAST NÄSTA

Strokehälsa - uppföljning efter stroke

Vardagliga aktiviteter

2. Har du svårare att klara dig själv i vardagliga aktiviteter efter din stroke? *(obligatorisk fråga)*

Här menas vardagliga aktiviteter som att duscha, klä på sig, laga mat eller att kunna ta sig ut.

- (•) Ja
- ( ) Nej
- ( ) Avstår från att svara

Har du svårigheter med att klä dig, tvätta dig eller duscha själv? *(obligatorisk fråga)*

- (•) Ja
- ( ) Nej

Har du svårigheter med att laga varm dryck eller mat? *(obligatorisk fråga)*

- ( ) Ja
- (•) Nej

Har du svårigheter med att ta dig ut? *(obligatorisk fråga)*

- (•) Ja
- ( ) Nej

Strokehälsa - uppföljning efter stroke

Gå och röra sig

3. Har du svårare att gå eller förflytta dig på ett säkert sätt efter din stroke? *(obligatorisk fråga)*

Besvär med att gå och röra sig kan öka risken för att ramla. Exempelvis när du förflyttar dig från säng till stol, i och ur bil eller i badrummet.

- ◯ Ja
- ◯ Nej
- ◯ Avstår från att svara

BAKÅT SPARA UTKAST NÄSTA

Strokehälsa - uppföljning efter stroke

Spasticitet

4. Upplever du en ökad stelhet i armar, händer och/eller ben efter din stroke? *(obligatorisk fråga)*

Spasticitet innebär en ökad spänningsgrad och stelhet i musklerna. Det kan leda till svårigheter att styra rörelser. Behandling av spasticitet kan förebygga smärta eller stelhet och förbättra möjligheterna till rehabilitering.

- ◯ Ja
- ◯ Nej
- ◯ Avstår från att svara

Strokehälsa - uppföljning efter stroke

Spasticitet

4. Upplever du en ökad stelhet i armar, händer och/eller ben efter din stroke? *(obligatorisk fråga)*

Spasticitet innebär en ökad spänningsgrad och stelhet i musklerna. Det kan leda till svårigheter att styra rörelser. Behandling av spasticitet kan förebygga smärta eller stelhet och förbättra möjligheterna till rehabilitering.

- (●) Ja
- ( ) Nej
- ( ) Avstår från att svara

Hindrar stelheten dig i vardagliga aktiviteter? *(obligatorisk fråga)*

Den ökade spänningen i musklerna kan till exempel göra det svårare att sköta hygien och påklädning.

- (●) Ja
- ( ) Nej

Sida 5 av 16

BAKÅT NÄSTA

Strokehälsa - uppföljning efter stroke

Smärta

5. Har det tillkommit någon ny smärta relaterat till din stroke? *(obligatorisk fråga)*

Efter en stroke kan smärta uppstå i olika delar av kroppen, men framförallt i den drabbade sidan. Smärta kan påverka många delar av livet och vara ett hinder i rehabiliteringen.

- ◯ Ja
- ◯ Nej
- ◯ Avstår från att svara

Strokehälsa - uppföljning efter stroke

Inkontinens

6. Har du mer problem att kontrollera din blåsa eller tarm efter din stroke? *(obligatorisk fråga)*

Efter en stroke kan man få problem med ofrivilligt läckage, att urinblåsan inte töms ordentligt eller svårt att hinna till toaletten i tid. Även tarmproblem kan förekomma såsom förstoppning och diarré.

- Ja
- Nej
- Avstår från att svara

BAKÅT

SPARA UTKAST

NÄSTA

Strokehälsa - uppföljning efter stroke

Kommunikation

7. Har du svårare att kommunicera med andra människor efter din stroke *(obligatorisk fråga)*

Att inte kunna tala eller göra sig förstådd kan vara ett hinder i många situationer. En del har svårt att uttrycka sig och förstå vad andra säger. Andra har svårt att uttala ord tydligt. Man kan även ha svårt att läsa och skriva.

- ◯ Ja
- ◯ Nej
- ◯ Avstår från att svara

Sida 8 av 16

BAKÅT SPARA UTKAST NÄSTA

Strokehälsa - uppföljning efter stroke

Sinnesstämning

8. Känner du mer oro eller nedstämdhet efter din stroke? *(obligatorisk fråga)*

Nedstämdhet, stresskänslighet, oro och humörsvängningar är vanligt efter stroke. Svårare tillstånd kan leda till depression vilket är ett medicinskt tillstånd som bör behandlas.

- ◯ Ja
- ◯ Nej
- ◯ Avstår från att svara

BAKÅT | SPARA UTKAST | NÄSTA

Strokehälsa - uppföljning efter stroke

Sinnesstämning

8. Känner du mer oro eller nedstämdhet efter din stroke? *(obligatorisk fråga)*

Nedstämdhet, stresskänslighet, oro och humörsvängningar är vanligt efter stroke. Svårare tillstånd kan leda till depression vilket är ett medicinskt tillstånd som bör behandlas.

- ◯ Ja
- ◯ Nej
- ◯ Avstår från att svara

Sida 9 av 16

BAKÅT SPARA UTKAST NÄSTA

Strokehälsa - uppföljning efter stroke

Sinnesstämning

8. Känner du mer oro eller nedstämdhet efter din stroke? *(obligatorisk fråga)*

Nedstämdhet, stresskänslighet, oro och humörsvängningar är vanligt efter stroke. Svårare tillstånd kan leda till depression vilket är ett medicinskt tillstånd som bör behandlas.

- Ja
- Nej
- Avstår från att svara

Sida 9 av 16

BAKÅT

SPARA UTKAST

NÄSTA

Strokehälsa - uppföljning efter stroke

Sinnesstämning

8. Känner du mer oro eller nedstämdhet efter din stroke? *(obligatorisk fråga)*

Nedstämdhet, stresskänslighet, oro och humörsvängningar är vanligt efter stroke. Svårare tillstånd kan leda till depression vilket är ett medicinskt tillstånd som bör behandlas.

- ◯ Ja
- ◯ Nej
- ◯ Avstår från att svara

BAKÅT | SPARA UTKAST | NÄSTA

Strokehälsa - uppföljning efter stroke

Kognition

9. Har du svårare att tänka, koncentrera dig eller att minnas efter din stroke? *(obligatorisk fråga)*

Vanliga svårigheter kan vara att komma igång, göra mer än en sak åt gången och lösa olika problem. Aktiviteter som kan vara utmanande är att läsa, betala sina räkningar och komma ihåg inbokade tider.

- Ja
- Nej
- Avstår från att svara

BAKÅT SPARA UTKAST NÄSTA

Strokehälsa - uppföljning efter stroke

Kognition

9. Har du svårare att tänka, koncentrera dig eller att minnas efter din stroke? *(obligatorisk fråga)*

Vanliga svårigheter kan vara att komma igång, göra mer än en sak åt gången och lösa olika problem. Aktiviteter som kan vara utmanande är att läsa, betala sina räkningar och komma ihåg inbokade tider.

- (•) Ja
- ( ) Nej
- ( ) Avstår från att svara

Begränsar detta dig från att delta i vardagliga aktiviteter? *(obligatorisk fråga)*

Man kan ha svårt att vara aktiv en hel dag och kan behöva vila eller ta pauser för att orka med.

- ( ) Ja
- ( ) Nej

BAKÅT   SPARA UTKAST   NÄSTA

1177 VÅRDGUIDEN

Tolvan Tolvansson
Inställningar | Logga ut

Start / Formulär / Strokehälsa - uppföljning efter stroke

Strokehälsa - uppföljning efter stroke

Livet efter stroke

10. Har du märkt av att saker som är viktiga för dig blivit svårare att genomföra efter din stroke? *(obligatorisk fråga)*

Här menas vad som är viktigt för dig, exempelvis fritid, arbete, sex och samliv samt att umgås med vänner och familj. Det kan vara lätt att dra sig undan och inte delta i samma aktiviteter som man gjorde innan sin stroke. Många upplever en ökad trötthet. Trötthet är ett vanligt symtom som kan ha flera olika orsaker.

- ◯ Ja
- ◯ Nej
- ◯ Avstår från att svara

Sida 11 av 16

BAKÅT | SPARA UTKAST | NÄSTA

Strokehälsa - uppföljning efter stroke

Familjerelationer

11. Har ditt förhållande till din familj blivit mer komplicerat eller stressfyllt efter din stroke? *(obligatorisk fråga)*

Livet förändras ofta efter en stroke såväl för dig som för dina närstående.

- ◯ Ja
- ◯ Nej
- ◯ Avstår från att svara

BAKÅT   SPARA UTKAST   NÄSTA

Strokehälsa - uppföljning efter stroke

Familjerelationer

11. Har ditt förhållande till din familj blivit mer komplicerat eller stressfyllt efter din stroke? *(obligatorisk fråga)*

Livet förändras ofta efter en stroke såväl för dig som för dina närstående.

- ( ) Ja
- ( ) Nej
- (•) Avstår från att svara

BAKÅT

NÄSTA

Strokehälsa - uppföljning efter stroke

Munhälsa

12. Har du svårare att sköta din munhygien efter din stroke? *(obligatorisk fråga)*

Det kan vara svårare att borsta sina tänder eller tandproteser. Försämrat tandstatus efter en stroke kan bero på flera olika orsaker och behöver utredas.

- ○ Ja
- ○ Nej
- ○ Avstår från att svara

Strokehälsa - uppföljning efter stroke

Äta och svälja

13. Har du svårare att äta eller svälja efter din stroke? *(obligatorisk fråga)*

En del har svårare att tugga och sätter lättare i halsen efter sin stroke. Exempelvis hostar mer än tidigare när man äter eller dricker. Ibland kan det vara svårt att få i sig tillräckligt med näring.

- ◯ Ja
- ◯ Nej
- ◯ Avstår från att svara

BAKÅT   SPARA UTKAST   NÄSTA

Strokehälsa - uppföljning efter stroke

Andra utmaningar

14. Har du några andra bekymmer efter din stroke, som påverkar din återhämtning? *(obligatorisk fråga)*

Konsekvenserna efter en stroke ser olika ut och kan variera över tid. Tveka inte att ta upp det som bekymrar dig vid ditt besök!

- ◯ Ja
- ◯ Nej
- ◯ Avstår från att svara

BAKÅT SPARA UTKAST NÄSTA

Strokehälsa - uppföljning efter stroke

Avslutning

Vill du berätta något mer inför ditt vårdbesök?

Antal tecken kvar: 250

Har du några rehabiliteringsinsatser just nu? *(obligatorisk fråga)*

Här menas om du får hjälp att anpassa träningen utifrån dina behov, med stöd av rehabiliteringspersonal. Det kan vara träning individuellt, i grupp alternativt självträningsprogram.

- (•) Ja
- ( ) Nej

Vem har besvarat detta frågeformulär? *(obligatorisk fråga)*

- (•) Jag själv ensam
- ( ) Jag själv med hjälp av anhörig/närstående eller vårdpersonal
- ( ) Endast närstående
- ( ) Annat

Vill du få mer information?

När du skickat in formuläret kommer du till en avslutningssida med länkar. Där kan du få tips om vad du kan ta upp på vårdbesöket. Du kan också delta i en forskningsstudie där vi utvärderar detta formulär via en webenkät.

Övriga kommentarer

Antal tecken kvar: 250

Sida 16 av 16

BAKÅT

NÄSTA

1177 VÅRDGUIDEN

Tolvan Tolvansson
Inställningar | Logga ut

Start / Formulär / Strokehälsa - uppföljning efter stroke

Strokehälsa - uppföljnir

Sammanställning

Läs igenom dina svar och bekräfta genom att klicka på knappen "Bekräfta c

Förhindra ny stroke

Fråga	Svar
1. Vill du få råd om vad du kan göra för att förhindra en ny stroke?	Ja

Vardagliga aktiviteter

Fråga	Svar
2. Har du svårare att klara dig själv i vardagliga aktiviteter efter din stroke?	Ja
Har du svårigheter med att klä dig, tvätta dig eller duscha själv?	Ja
Har du svårigheter med att laga varm dryck eller mat?	Nej
Har du svårigheter med att ta dig ut?	Ja

Gå och röra sig

Fråga	Svar
3. Har du svårare att gå eller förflytta dig på ett säkert sätt efter din stroke?	Ja

Spasticitet

Fråga	Svar

Andra utmaningar

Fråga	Svar
14. Har du några andra bekymmer efter din stroke, som påverkar din återhämtning?	Ja

Avslutning

Fråga	Svar
Vill du berätta något mer inför ditt vårdbesök?	*Frågan är ej besvarad*
Har du några rehabiliteringsinsatser just nu?	Ja
Vem har besvarat detta frågeformulär?	Jag själv ensam

Vill du få mer information?

Fråga	Svar
Övriga kommentarer	*Frågan är ej besvarad*

BAKÅT

BEKRÄFTA DINA SVAR

Tack för dina svar!

Vad kan jag göra själv och hur kan jag få stöd?

Det är mycket du kan göra själv, men ibland kan du behöva stöd från andra. Här kan du läsa mer så att du känner dig förberedd och kan vara delaktig i din vård. Följ länken nedan:

Läs mer, tips och råd

Deltagande i en forskningsstudie

För att kunna utvärdera och förbättra Strokehälsa görs en studie med en webbenkät som tar ca 10 minuter. Klicka på länken om du vill delta och få mer information om studien.

Studieinformation och webbenkät

Strokehälsa har skapats med en av 1177 Vårdguidens e-tjänster, Formulärhantering. Frågorna i Strokehälsa har utvecklats vid Göteborgs universitet i samverkan med Strokeföreningen. På 1177.se finns bra information om stroke om du vill veta mer. Kom ihåg att du kan komma tillbaka till Strokehälsa igen via 1177 (under "Alla övriga tjänster" och "Fyll i formulär") om du vill se dina svar innan besöket.

TILL 1177 INKORG   TILL MINA FORMULÄR

Example of the summary view shown to health professionals, within the secure patient portal

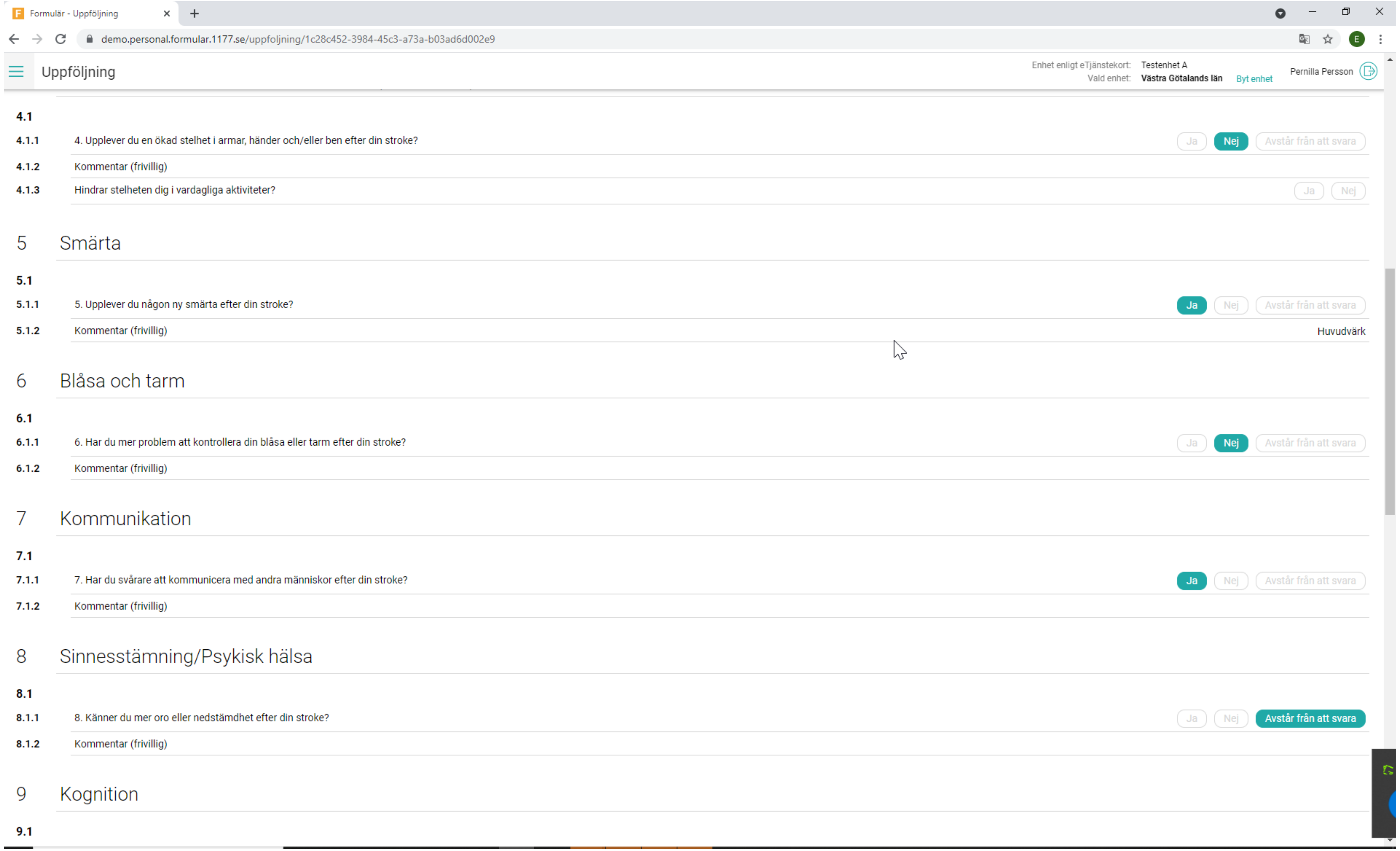

This summary is based on version 1.1.

Advisory texts

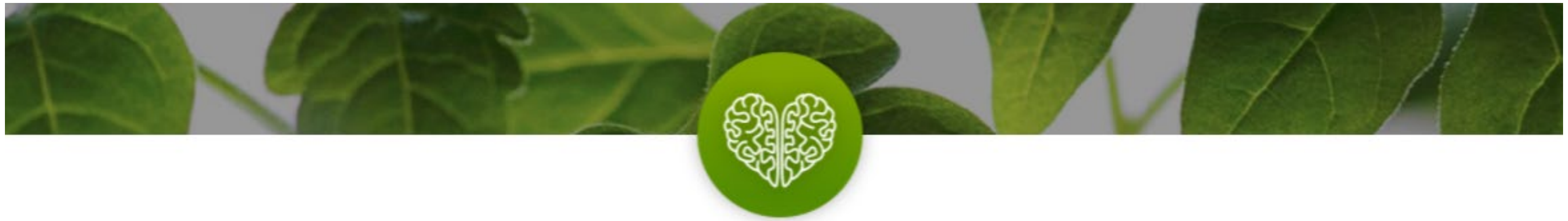

Vad kan jag göra själv och hur kan jag få stöd?

Diskutera gärna med din vårdgivare om hur dina vårdkontakter ska se ut i framtiden. Du kan även be om råd och få information om hur du kan komma i kontakt med andra vårdgivare.

Genom att läsa denna information kan du förbereda dig inför ditt vårdbesök. Fundera på vad du kan göra själv och om du behöver stöd.

FORTSÄTT

Vad kan jag göra själv och hur kan jag få stöd?

1. Förhindra ny stroke

Att insjukna på nytt är en rädsla många bär på. Risken för en ny stroke kan minskas betydligt genom en kombination av livsstilsförändringar, medicinering och blodtryckskontroller. Din läkare eller sjuksköterska kan hjälpa dig att lägga upp en plan utifrån dina behov.

TILLBAKA NÄSTA

Vad kan jag göra själv och hur kan jag få stöd?

2. Vardagliga aktiviteter

Det är bra för dig att återuppta aktiviteter du gjort tidigare. Du kan få stöd av en arbetsterapeut med träning och hjälpmedel för att klara vardagen. Du kan också få hjälp att göra aktiviteter på ett nytt sätt så att du kan göra det som är viktigt för dig.

Vad kan jag göra själv och hur kan jag få stöd?

3. Gå och röra sig

Förbättringar kan ske under lång tid efter en stroke. Du kan träna gång och balans för att röra dig lättare och minska risken att falla. Diskutera träning och behov av hjälpmedel med fysioterapeut eller rehabteamet.

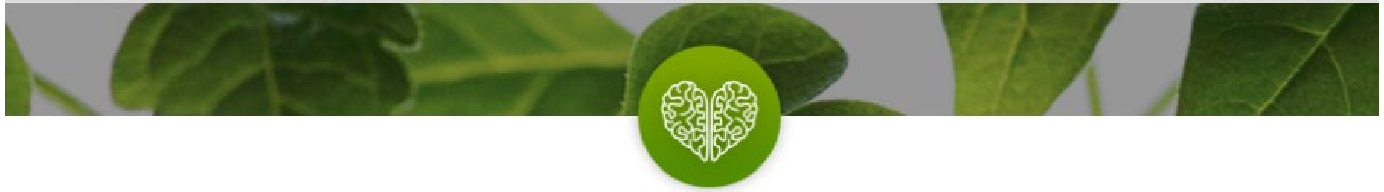

Vad kan jag göra själv och hur kan jag få stöd?

4. Spasticitet

Rådgivning och behandling av spasticitet kan förebygga smärta, stelhet och förbättra möjligheterna till rehabilitering. En fysioterapeut eller arbetsterapeut kan bedöma och föreslå åtgärder. Din läkare kan hjälpa dig med vidare utredning och föreslå lämpliga åtgärder.

TILLBAKA N[illegible]TA

Vad kan jag göra själv och hur kan jag få stöd?

5. Smärta

Oavsett var smärtan sitter är det viktigt att den utreds och om möjligt behandlas. En fysioterapeut kan bedöma om smärtan beror på besvär från muskler eller leder och hjälpa dig med åtgärder. Din läkare kan också hjälpa dig att utreda och föreslå lämpliga åtgärder.

Vad kan jag göra själv och hur kan jag få stöd?

6. Inkontinens

Besvär med att hålla sig och att hinna till toaletten i tid är vanligt efter stroke. Även tarmproblem, som förstoppning och diarré, kan förekomma. Det finns inkontinenshjälpmedel och behandling som kan underlätta vardagslivet. Du kan få hjälp av en sjuksköterska med särskild kunskap om inkontinensbesvär efter stroke. Ta kontakt med din vårdcentral om du vill få mer information.

TILLBAKA NÄSTA

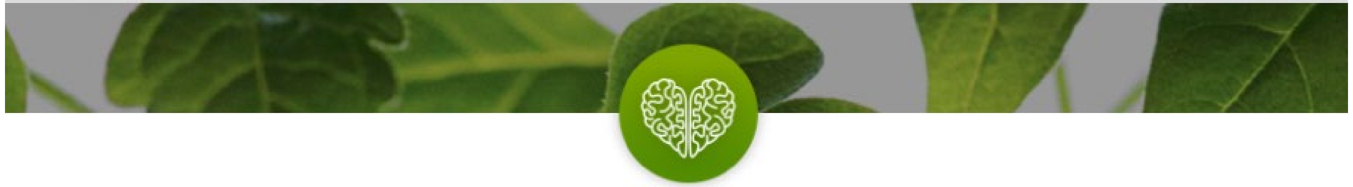

Vad kan jag göra själv och hur kan jag få stöd?

7. Kommunikation

Talet förbättras ofta över tid och man lär sig sätt att hantera detta i vardagen. Ofta är det lättare att prata med en person i taget. En logoped kan utreda, behandla, stötta vid anpassningar och prova ut kommunikationshjälpmedel.

TILLBAKA NÄSTA

Vad kan jag göra själv och hur kan jag få stöd?

8. Sinnesstämning

Vardagliga rutiner, promenader och att berätta för någon om hur du mår kan hjälpa dig att må bättre. Har du stora besvär ska du inte vänta med att söka hjälp. Tala med din läkare, sjuksköterska eller psykolog. Besvären kan lindras med medicinsk behandling och samtalsstöd. Känns det akut så kontaktar du sjukvårdsupplysningen, 1177 eller 112.

TILLBAKA NÄSTA

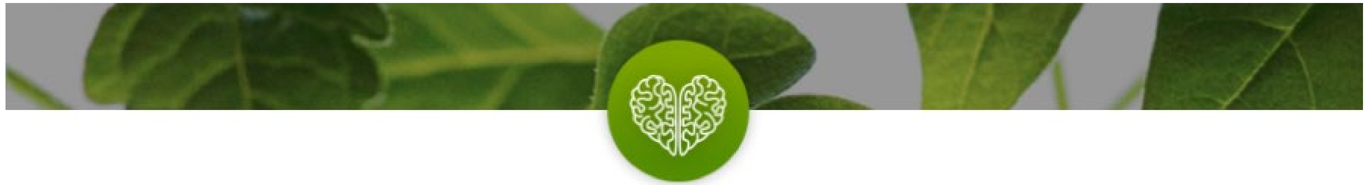

Vad kan jag göra själv och hur kan jag få stöd?

9. Kognition

Problem med exempelvis minne och koncentration märks ofta när man återgått till sina vardagliga rutiner. En lugn miljö och att bara göra en sak i taget kan underlätta. Du kan hitta nya sätt att göra aktiviteter på och använda tekniska hjälpmedel för att bättre hantera din livs-situation. Du kan ta upp detta med rehabteamet eller din läkare som kan hänvisa vidare.

TILLBAKA NÄSTA

Vad kan jag göra själv och hur kan jag få stöd?

10. Livet efter stroke

Du vet själv bäst hur du behöver leva för att må bra. Ibland hittar man nya intressen efter sin stroke. Delta gärna i en stödgrupp eller en patientförening exempelvis Stroke-Riksförbundet, Afasiförbundet eller Neuroförbundet. Det kan vara till hjälp att se att du och din familj inte är ensamma.

Om du planerar att återgå i arbete kan en rehab-koordinator eller din läkare hjälpa dig med fortsatt planering och relevanta kontakter. Om du har stora besvär med trötthet kan du behöva en individuell bedömning för att ta reda på orsaken. Rehabteamet kan hjälpa dig att hitta sätt för att underlätta din vardag.

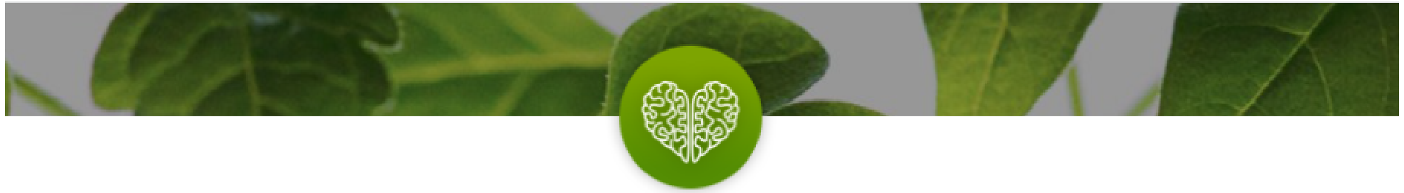

Vad kan jag göra själv och hur kan jag få stöd?

11. Familjerelationer

Det finns anhörigstödjare i alla kommuner som kan vara ett bra stöd. Genom att delta i en stödgrupp kan du och din familj träffa andra i liknande situation och se att ni inte är ensamma. Även patient-föreningar kan vara ett bra stöd exempelvis Stroke-Riksförbundet, Afasiförbundet eller Neuroförbundet. Ibland kan närstående följa med vid ett vårdbesök för att få information och svar på sina frågor.

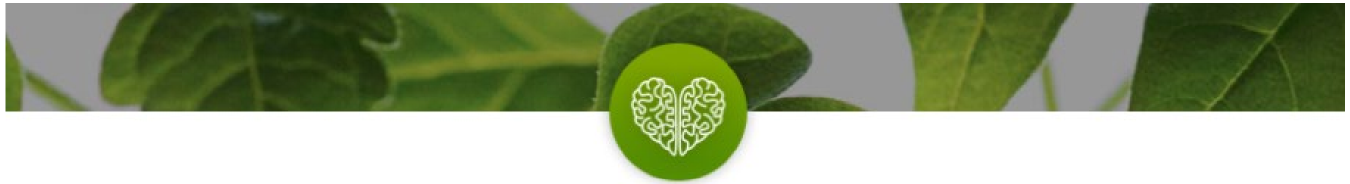

Vad kan jag göra själv och hur kan jag få stöd?

12. Munhälsa

Risken för karies kan öka efter en stroke. Du kan förebygga karies genom en god munhygien. Det är bra att boka en tid hos tandläkaren några månader efter en stroke. Om du har besvär med din munhälsa kan du efterfråga särskilt tandvårdsstöd.

TILLBAKA NÄ[illegible]TA

Vad kan jag göra själv och hur kan jag få stöd?

13. Äta och svälja

Det är viktigt att få i sig näring för att kunna återhämta sig på ett bra sätt. Du kan få bra råd av din sjuksköterska och hjälp att hänvisas vidare vid behov. Om du har svårt att svälja kan logopeden göra en bedömning och ge råd. Med underlättande teknik och anpassningar kan sväljningen göras säkrare.

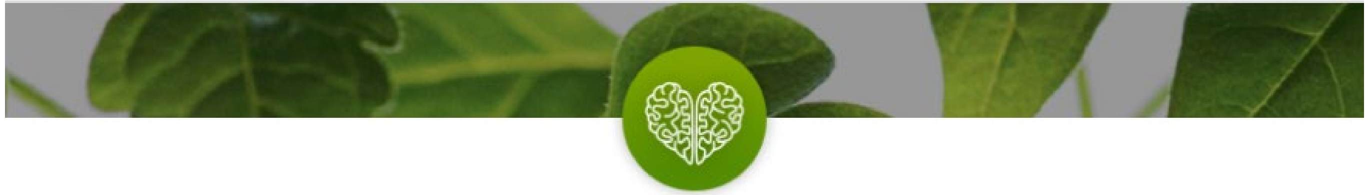

Vad kan jag göra själv och hur kan jag få stöd?

14. Andra utmaningar

Tveka inte att ta upp det som bekymrar dig! Människor drabbas på olika sätt av sin stroke. Ofta kan problemen vara dolda för omgivningen. Du kan få bra tips och råd via en patientförening. Kontakta din vårdcentral eller rehabteam så de kan göra en bedömning och ge dig stöd.

TILLBAKA NÄSTA

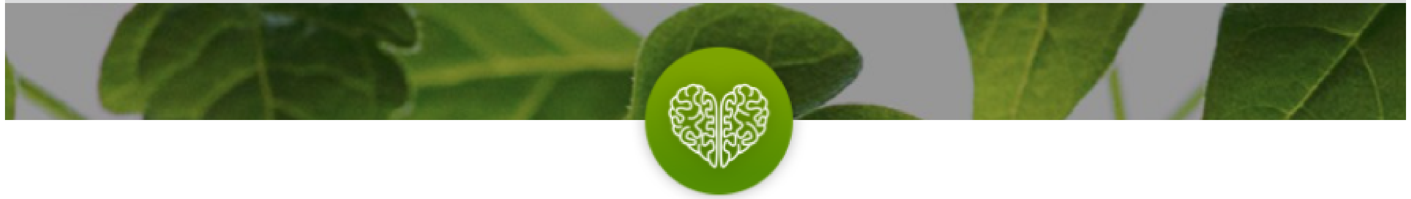

Vad kan jag göra själv och hur kan jag få stöd?

Med rätt stöd kan du ta medansvar för din hälsa

Din vårdgivare har generell kunskap om stroke, men du är expert på dina hälsoproblem och din situation.

Tillsammans med vårdgivare, närstående och andra viktiga stödpersoner kan du vara delaktig och ha medansvar för din hälsa.

Om du har en vårdplan från en tidigare vårdgivare kan du gärna ta med den till besöket. Genom att du delar med dig av dina tankar kan vårdgivaren hjälpa dig att anpassa vården efter dina behov.

GÅ TILLBAKA OCH LÄS IGEN

Eller stäng fönstret alternativt fliken för att återgå till 1177.
